# Comparison of SERS pH probe responses after microencapsulation within hydrogel matrices

**DOI:** 10.1117/1.JBO.26.9.097001

**Published:** 2021-09-13

**Authors:** Dayle Kotturi, Sureyya Paterson, Mike McShane

**Affiliations:** a Texas A&M University, Department of Biomedical Engineering, College Station, Texas, United States; b Texas A&M University, Department of Materials Science and Engineering, College Station, Texas, United States

**Keywords:** surface-enhanced Raman spectroscopy, pH sensing, continuous monitoring, implantable, biocompatible, hydrogel, mercaptobenzoic acid

## Abstract

**Significance:** Personalized medicine requires the tracking of an individual’s metabolite levels over time to detect anomalies and evaluate the body’s response to medications. Implanted sensors offer effective means to continuously monitor specific metabolite levels, provided they are accurate, stable over long time periods, and do no harm.

**Aim:** Four types of hydrogel embedded with pH-sensitive sensors were evaluated for their accuracy, sensitivity, reversibility, longevity, dynamic response, and consistency in static versus dynamic conditions and long-term storage.

**Approach:** Raman spectroscopy was first used to calibrate the intensity of pH-sensitive peaks of the Raman-active hydrogel sensors in a static pH environment. The dynamic response was then assessed for hydrogels exposed to changing pH conditions within a flow cell. Finally, the static pH response after 5 months of storage was determined.

**Results:** All four types of hydrogels allowed the surface-enhanced Raman spectroscopy (SERS) sensors to respond to the pH level of the local environment without introducing interfering signals, resulting in consistent calibration curves. When the pH level changed, the probes in the gels were slow to reach steady-state, requiring several hours, and response times were found to vary among hydrogels. Only one type, poly(2-hydroxyethyl methacrylate) (pHEMA), lasted five months without significant degradation of dynamic range.

**Conclusions:** While all hydrogels appear to be viable candidates as biocompatible hosts for the SERS sensing chemistry, pHEMA was found to be most functionally stable over the long interval tested. Poly(ethylene glycol) hydrogels exhibit the most rapid response to changing pH. Since these two gel types are covalently cross-linked and do not generally degrade, they both offer advantages over sodium alginate for use as implants.

## 1 Introduction

Personalized medicine has been predicted to be disruptive, eventually upending the medical industry by shifting expenditures from reactionary to preventative care.^1^ Continuous glucose monitoring devices, such as the Eversense by Senseonics, the G6 by Dexcom, and the Freestyle Libre by Abbott, have been on the leading edge of this effort by transforming the management of diabetes.^2^ By developing sensors that can detect metabolites other than glucose, this technology can potentially be extended to manage other chronic conditions, such as kidney disease, Parkinson’s, Alzheimer’s, and cancer.^3^^–^^5^ Further, while controversial, there is interest from generally healthy individuals to use such tools for managing wellness, optimizing fitness, etc.

Expanding the set of tools to enable tracking a range of metabolites and storing these values in personal electronic medical and other accessible records is the first step.^6^ For convenience, comfort, and value, such devices must enable frequent, facile sampling and possess a long operating lifetime, which is especially challenging for implanted devices.^7^ Optical methods offer promise for minimally invasive monitoring but must be built on stable, reversible transduction chemistry, and biocompatible packaging. The two main branches of optical techniques are photon absorption and photon scattering. Within the scattering modalities, there are both elastic (Rayleigh) and inelastic (Raman) techniques. Fluorescence-based sensors detect photons re-emitted at lower energies after absorption by special chromophores, and typically lose sensitivity over time due to photobleaching.^8^ In contrast, Raman spectroscopy (RS) offers potential to extend the operating lifetime of a sensor, by working at a lower energy which does not degrade the materials by photobleaching. Further, the portion of the emitted light that is inelastically scattered after the interaction encodes unique molecular “fingerprints” and allows the possibility of multi-metabolite detection. Unfortunately, RS is not sensitive. The very low efficiency of Raman inelastic scattering (compared to elastic scattering) limits its usefulness.

Fortunately, the intrinsically weak Raman signals can be amplified using a technique called surface-enhanced Raman spectroscopy (SERS).^9^ SERS has commonly been used to amplify signals in a range of assay configurations, from single-shot solution phase using plasmonic nanoparticles (NPs)^10^^–^^13^ to some limited continuous monitoring cases using solid substrates.^14^^–^^19^ Several recent reviews describe the advancement of both these types of assays.^20^^–^^23^ With respect to continuous monitoring, solid substrates offer reliable and repeatable sensing properties while NPs must be prevented from migrating away from the insertion site for repeatably valid readings. This problem has been addressed by constraining the NPs within the matrix of various materials. For biomedical application such as continuous *in vivo* monitoring, biocompatible gels have been employed^24^ and additional benefits have been observed from this approach. First, the useful lifetime is extended because the gel prevents NP movement while blocking large molecules such as proteins that may foul the NPs.^24^^–^^30^ Second, the foreign body response to the sensing chemistry is attenuated by isolating the NPs from the cells responsible for the immune/inflammatory response while still allowing the analyte(s) of interest to permeate and interact appropriately.^26^ Of course, the encapsulating matrix must not introduce substantial intrinsic Raman signals that would interfere or otherwise confound the measurement.

In this study, pH is used primarily as a model target analyte. While low values of pH in interstitial fluid may be an indicator of insulin resistance,^31^^,^^32^ as well as inflammation and solid tumors,^33^ the focus of this work is not on pH as a primary biomarker. Rather, the focus is on evaluating the ability of the hydrogels to encapsulate the probes and to allow the SERS interaction with the probes to occur under different conditions. Note also that our method of encapsulating and embedding the SERS-active material can be applied to detect a range of analytes, for example, by adding enzymes.^29^

The same pH-sensitive material, mercaptobenzoic acid gold nanoparticles (MBA AuNPs), is encapsulated within polyelectrolyte microcapsules (MCs) and then embedded in four candidate hydrogels. The hydrogels comprise materials used in biocompatible implants. The MCs and the hydrogel serve as two layers of anti-fouling protection for the MBA AuNPs, as well as constraining their location while still allowing localized NP aggregation and dissociation. This study expands on a preliminary study in which a small sample size was evaluated over a shorter timescale, in which some interesting trade-offs in responses were observed.^34^

The hydrogels are evaluated for their ability to allow the pH-sensitive SERS assays to reliably reflect the pH level of the dynamic and static pH environments over extended times. They are evaluated by comparing the SERS intensity of known MBA Raman peaks in the categories of reversibility, responsiveness, range (the extent to which the Raman intensities change in response to a span of pH environments), sensitivity, longevity (how closely aged gels match the Raman intensities of new gels), and consistency (how closely the Raman intensities match under static and dynamic pH conditions). To our knowledge, this is the first time that different biomaterials for encapsulating SERS reporters have been evaluated; further, it appears this is the first case of determining the rate of real-time reversible response by tracking the MBA peak amplitudes as they react to a change in environmental pH and achieve steady-state.

## 2 Experimental

### 2.1 Materials

Gold chloride trihydrate, sodium citrate trihydrate, hydroxylamine hydrochloride, sodium carbonate, sodium bicarbonate, poly(diallyldimethylammonium chloride) (PDADMAC, average molecular weight 100 to 200 kDa), poly(sodium 4-styrenesulfonate) (PSS, average molecular weight 70 kDa), 4-MBA, methanol, and ethylenediaminetetraacetic acid, 2-(n-morpholino) ethanesulfonic acid (MES) sodium salt, alginic acid sodium salt (from brown algae, ∼250  cps for 2% solution at 25°C), calcium carbonate (${\mathrm{CaCO}}_{3}$), triethanolamine (TEOA), and 2-dimethoxy-2-phenyl-acetophenone (DMPAP) were obtained from Sigma-Aldrich (St. Louis, Missouri). Ethanol (200 proof, USP) and calcium chloride (${\mathrm{CaCl}}_{2}$) were purchased from Decon Labs and Macron Fine Chemicals (Center Valley, Pennsylvania), respectively. 2-Hydroxyethyl methacrylate (HEMA, ophthalmic grade) and tetraethylene glycol dimethacrylate were purchased from Polysciences, Inc. (Warrington, Pennsylvania). Four-arm poly(ethylene glycol)-thiol (MW 10,000) was obtained from Laysan Bio, Inc. (Arab, Alabama) and 4-arm poly(ethylene glycol)-vinyl sulfone (MW 10,000) was obtained from JenKem Technology (Plano, Texas).

### 2.2 Instrumentation

#### 2.2.1 UV–Vis spectroscopy

Absorbance spectra were collected with a Cary 50 UV–Vis spectrophotometer (Agilent Technologies, Santa Clara, California). UV–Vis extinction was recorded in the range of 400 to 800 nm, a scan speed of 300  nm/s, and 0.5-nm resolution.

#### 2.2.2 Raman spectroscopy

Raman scattering spectral data were collected with a Raman spectrometer WP785 (Wasatch Photonics, Morrisville, North Carolina) with external 785-nm laser source (Innovative Photonic Solutions, Monmouth Junction, New Jersey) measured at the sample as 23.5 mW. Integration time for each raw measurement was 1000 milliseconds (ms). This integration time was determined to be the minimum integration time needed for the intensity of the $1430\text{-}{\mathrm{cm}}^{-1}$ peak to be at least 20% of the reference peak intensity in neutral pH. Because of its pH-insensitivity, the $1582\text{-}{\mathrm{cm}}^{-1}$ peak was used as the intensity reference to normalize the spectra.

### 2.3 Methods

#### 2.3.1 NP formation and functionalization

All glassware were cleaned with Aqua regia prior to use. AuNPs were synthesized using a seed-mediated growth method.^35^ Briefly, 335  μl of 25 mM sodium tetrachloroaurate was added to a 50 ml boiling solution of 2.2 mM sodium citrate in a three-neck round bottom flask. After 15 min, the temperature was reduced to 90°C and left to stir for 1 h. About 670  μl of the resultant solution was subsequently removed for UV–Vis characterization and an additional 335  μl of 60 mM sodium citrate was added, followed by further addition of 335  μl of 25 mM sodium tetrachloroaurate, this was then left to stir for an additional 30 min. The 670  μl of NP solution between each layer step was used for UV–Vis characterization to determine the final layer number. For these measurements, a 1 in 5 dilution of the NP solution in deionized (DI) water was characterized and the layer addition was stopped when the absorption peak reached a $\lambda_{\max}$ of 530 nm. Typically, we characterize these materials using UV–Vis spectroscopy, dynamic light scattering, transmission electron microscopy, and/or nanoparticle tracking analysis to verify the size, shape, and concentration of the AuNPs, as described in previous publications.^29^^,^^36^ Since we followed the established synthesis protocols used in previous work, only UV–Vis was used in this case to verify the expected absorption peak (530 nm) corresponding to an NP diameter of 54 nm.

The detection of pH is accomplished by functionalizing the resultant AuNPs with MBA. The pH-sensitivity of MBA is well known.^29^^,^^37^^–^^47^ MBA has two strong peaks due to ring breathing at 1072 and $1582\;{\mathrm{cm}}^{-1}$, which can be considered pH-insensitive and two smaller peaks at $1430\;{\mathrm{cm}}^{-1}$ (COO- stretching) and $1702\;{\mathrm{cm}}^{-1}$ (C=O stretching) whose intensity changes with local pH.

The functionalization of MBA was done using established methods.^48^ Briefly, 200  μl of 10 mM of MBA (in 200 proof ethanol) was added to 1800  μl of 1.1 nM AuNPs and shaken lightly (using a vortex on the shaking action) for 5 min. The NPs were then centrifuged at 6000 g for 15 min and the pellet was removed and resuspended in 400  μl of DI water to remove excess MBA.

#### 2.3.2 Encapsulation

The pH-responsive NPs were encapsulated into polyelectrolyte multilayer (PEM) MCs using a co-precipitation method described previously.^49^ Briefly, 400  μl of the MBA-functionalized NPs from Sec. 2.3.1 was added to 8 ml of 250 mM sodium carbonate solution under vigorous stirring. Subsequently, 8 ml of 250 mM calcium chloride was quickly added to the mixture to form ${\mathrm{CaCO}}_{3}$ microparticles (MPs). The suspension was then stirred for an additional 30 s before standing for 10 min. The sedimented MPs were collected through centrifugation at 1000 g for 30 s and further cleaned with 1 ml of 5 mM, pH 8.0 sodium bicarbonate. The MPs were then coated with a PEM using a layer-by-layer method. This was achieved through alternating layers of PDADMAC and PSS (each polyelectrolyte was dissolved in 5 mM sodium bicarbonate buffer at a concentration of 20  mg/ml). The MPs were rinsed with 5 mM sodium bicarbonate between each polyelectrolyte deposition until a total of 20 layers of alternating PDADMAC/PSS were achieved. The MCs were formed by immersing the MP solution into 15 ml of 0.2 mM, pH 6.1 MES buffer. This was repeated three times to achieve a full dissolution of the ${\mathrm{CaCO}}_{3}$ core. The MCs were then washed with sodium bicarbonate and stored in ${\mathrm{dH}}_{2}O$ for further use in hydrogels, and dry weights were calculated.

#### 2.3.3 Hydrogel fabrication

MCs were embedded in hydrogels to create “pseudo-solid-state” materials that can be physically manipulated. Trapping the capsules in hydrogels maintains a fixed relative position of the capsules in 3D space while allowing hydration and small molecule access to the embedded capsules. Here, several different hydrogels were explored to evaluate their suitability for this purpose. To keep consistent concentrations of MCs within the hydrogel, each hydrogel contained 3.65 mg of MCs (taken from dry weights).

##### Alginate

To form the alginate hydrogel, 75  μl (3.65 mg) of MCs and 25  μl of ${\mathrm{CaCO}}_{3}$ (46.1  mg/ml) were added to 200  μl of 3% w/v of alginic acid. The ${\mathrm{CaCO}}_{3}$ provides a source of calcium for faster gelation. This solution was then mixed with 100  μl of 0.5 M, pH 5.8 MES buffer and added into a glass mold consisting of two glass slides sandwiched between a 0.2-μm-thick Teflon spacer. The hydrogel was left in the mold for 15 min whereby it was then transferred to a fresh solution of 10 mM Tris buffer (pH 7.4) containing 10 mM of ${\mathrm{CaCl}}_{2}$ for storage.

##### Poly(ethylene glycol)

A poly(ethylene glycol) vinyl sulfone-thiol (PEG VS-SH) click chemistry gel was formulated by mixing a 1:1 ratio of 200  μl of 4-arm PEG vinyl-sulfone and 200  μl of 4-arm PEG thiol together with 75  μl (3.65 mg) of MCs with 80  μl TEOA (100 mM). To this solution, 245  μl of PBS was added and the precursor solution was transferred to a silicone rubber mold. For long-term storage, the gel was stored in 10 mM Tris buffer (pH 7.4) for further use.

##### Poly(2-hydroxyethyl methacrylate)

The poly(2-hydroxyethyl methacrylate) (pHEMA) gel was formed by adding 2.5 mg of DMPAP to 250  μl of pHEMA. This was vortexed for 5 min to dissolve the DMPAP. To this mixture, 5  μl of TEGMA and 90  μl of ethylene glycol were added and vortexed again for a further 2 min. About 55  μl of MCs (containing 3.65 mg) was added and the mixed solution was pipetted into a glass mold as per the alginate gel. This was then cured under a UV lamp for 10 min with frequent turning every 30 s. The gel was stored in 10 mM Tris buffer (pH 7.4) for further use.

##### Poly(2-hydroxyethyl methacrylate) co-acrylamide

The poly(2-hydroxyethyl methacrylate) co-acrylamide (pHEMA-coA) gel was formed by adding 2.5 mg of DMPAP to 187.5  μl of pHEMA. This was vortexed for 5 min to dissolve the DMPAP. About 62.5  μl of acrylamide was then added to this as well as 5  μl of TEGMA and 90  μl of ethylene glycol. The mixture was vortexed again for a further 2 min. About 55  μl of MCs (containing 3.65 mg) was added and the mixed solution was pipetted into a glass mold as per the alginate gel. This was then cured under a UV lamp for 10 min with frequent turning every 30 s. The gel was then stored in 10 mM Tris buffer (pH 7.4) for further use.

#### 2.3.4 Buffer solutions

As-formed hydrogel samples were stored in buffers until used for testing, and in between tests for the long-term studies. The choice of buffers for storage and testing depended on the hydrogel characteristics. For alginate, the buffer used was 1 mM Tris with 10 mM ${\mathrm{CaCl}}_{2}$. ${\mathrm{CaCl}}_{2}$ was needed to stabilize the ionically cross-linked alginate.^50^ For PEG, pHEMA, and pHEMA-coA, the buffer used was 1 mM Tris. No cross-linking stabilization was needed for these hydrogels since they are covalently cross-linked, therefore ${\mathrm{CaCl}}_{2}$ was omitted.

While the pH of interstitial fluid ranges from 6.6 to 7.6,^32^ we used a broader acidic range. For the static measurements, eight 1-l solutions were titrated to pH 4 to 7.5 in steps of 0.5. This pH range was chosen based on our desire to broadly characterize pH with Raman intensity over a range that included body pH levels and also considered the reduced sensitivity of MBA in the alkaline range. For the dynamic measurements, three 1-l solutions were titrated to pH 4, 7, and 10. We used extreme values because this is the first time we are testing the sensing material in dynamic conditions.

#### 2.3.5 Static measurements

Hydrogel samples were stored as cross-linked films in petri dishes immersed in pH 7.4 buffer solution ($\text{Tris}/{\mathrm{CaCl}}_{2}$ for alginate; Tris for all other types) at 4°C. Prior to spectroscopic analysis, a 6-mm biopsy punch was used to extract discs from the hydrogel slab. Each disc was rinsed 3 times and then soaked for 1 h in buffer solution ranging from pH 4.0 to 7.5, in steps of 0.5. After washing, the discs were transferred to a quartz slide with 30  μl of the buffer pipetted onto the sample surface (for hydration). Initially, a Raman spectrum was collected with the laser off and the room dark. This “dark” spectrum was subtracted from each raw spectrum prior to averaging. Next, five raw Raman spectra were collected using 23.5-mW laser power (measured at the sample) and 1000-ms integration time and then averaged to a single “measurement” to reduce random signal noise in the spectrum. Five measurement scans were then taken for each hydrogel disc. This process was repeated for five different discs of each type of hydrogel at eight different pH levels. Note that both the dark and raw spectra were archived to disk with automatically generated, unique file names based on the date and time of acquisition and that each measurement was saved to disk using its date and time of calculation to form its unique file name.

#### 2.3.6 Dynamic measurements

**Fig. 1:**
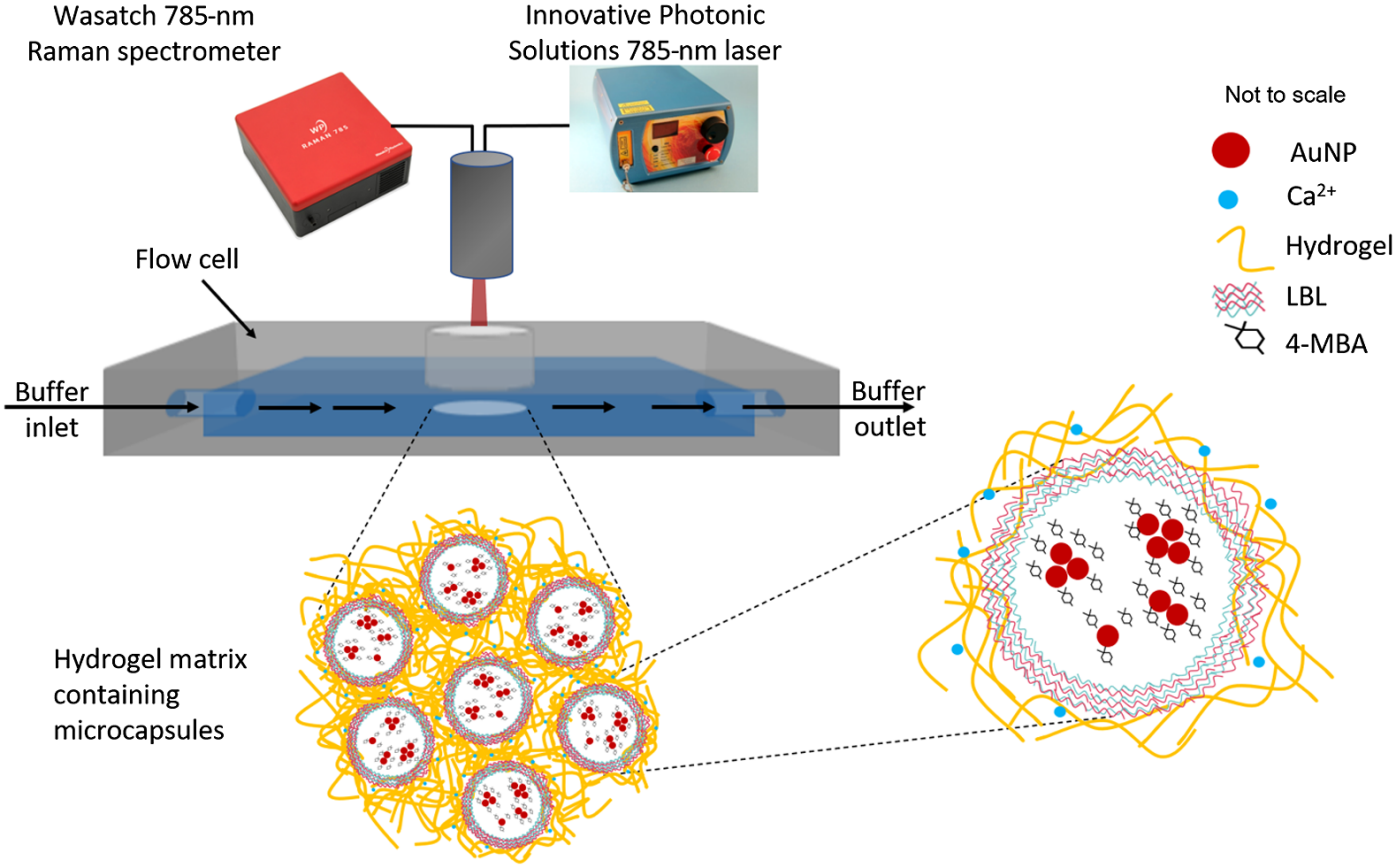
Apparatus for dynamic measurements. The AuNPs are functionalized with pH sensing material, MBA, encapsulated in MCs, embedded in hydrogel, and then fixed in place inside the flow cell so that it is visible to the laser and detector.

Hydrogel samples were stored as cross-linked films in petri dishes immersed in pH 7.4 buffer solution at 4°C. Prior to spectroscopic analysis, a 6-mm biopsy punch was used to extract a disc from the hydrogel slab, which was then transferred to a flow cell (Fig. 1). The disc was clipped to the quartz slide so that it would remain in place as the buffer pumped through the chamber. The clips were made from 1-mm-thick rubber sheet, excised as a ring shape using 6- and 4-mm biopsy punches, cut in half and glued to the quartz slides, off from the center of the flow cell window to avoid interaction with the laser light. The flow cell was sealed watertight with 12 bolts, washers, and wing nuts, pressure-fitting the two halves of the flow cell against a rubber gasket in contact with the quartz. The sample was visible through a window made by the quartz slide in the flow cell. Buffer was constantly pumped through the flow cell at the rate of 4  ml/min for the duration of the experiment. There was no difference between the rate of the buffer entering versus leaving the flow cell; it flowed through continuously as shown by the inlet and outlet arrows in Fig. 1. Positioning the probe at the focal length over the sample, spectra were recorded approximately every 2 min for approximately 2 h at a single pH level. At the end of the interval, the solution was changed to another pH. The same order of pH values, pH=7, 4, 10, 7, 10, 4, 10, 7, and 4, was used for all of the flow cell series. For each gel type, three flow cell series were completed, using a new hydrogel disc sample each time.

#### 2.3.7 Analysis

The analysis for both the static and dynamic sensing began using the “measurements” defined in Sec. 2.3.5. These average spectra were baseline-corrected via an asymmetric least-squares method,^51^ and then normalized by an average of the five nearest values to the intensity at wavenumber $1582\;{\mathrm{cm}}^{-1}$. Since this average did not necessarily match the local maximum, the maximum of the normalized spectrum did not necessarily match 1. For the static sensing, the measurements of all five punches of a single gel type were averaged and the standard deviations were determined at each pH level. The standard deviation was plotted as the error bars of the normalized intensity, and the color and shape of the symbols were used to indicate the gel type. For the dynamic sensing, the normalized intensities at 1430 and $1702\;{\mathrm{cm}}^{-1}$ were extracted from the spectra and plotted versus time.

## 3 Results and Discussion

### 3.1 Verification of Sensing Material

In preparation for the evaluation of the sensing material in both static and dynamic pH environments, the functionalization of the sensing materials was first verified with RS by making measurements at 10 milestones in the fabrication process: (1) MBA on AuNPs, (2) MBA AuNPs into ${\mathrm{NaCO}}_{3}$, (3) MBA AuNPs with ${\mathrm{NaCO}}_{3}$ and ${\mathrm{CaCl}}_{2}$, (4) first wash with ${\mathrm{NaCO}}_{3}$, (5) after first bilayer PDADMAC/PSS, (6) after five bilayers, (7) after 10 bilayers, (8) after first MES buffer wash, (9) after third MES buffer wash and into 10 mM pH 7, and (10) in alginate gel at pH 7. At each milestone, the Raman spectra were evaluated for the detectability of the expected MBA Raman peaks and signal quality (i.e., peak intensity >> noise).

**Fig. 2:**
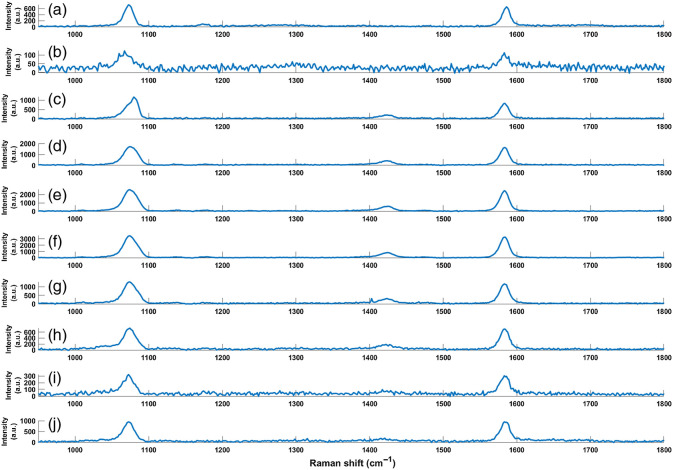
Raman averaged, baseline-corrected spectra at each step of alginate hydrogel fabrication. Steps are (a) MBA on AuNPs, (b) MBA AuNPs into ${\mathrm{NaCO}}_{3}$, (c) MBA AuNPs with ${\mathrm{NaCO}}_{3}$ and ${\mathrm{CaCl}}_{2}$, (d) first wash with ${\mathrm{NaCO}}_{3}$, (e) after first bilayer PDADMAC/PSS, (f) after five bilayers, (g) after 10 bilayers, (h) after first MES buffer wash, (i) after third MES buffer wash and into 10 mM pH 7, and (j) in alginate gel at pH 7.

Each step of the fabrication process consists of seven sub-steps related to acquisition and processing (described in Sec. 2.3.5), with results included in the Supplementary Material (Figs. S1-S10). The most important observations from these measurements are that the characteristic MBA peaks (at 1072 and $1582\;{\mathrm{cm}}^{-1}$) are evident throughout the stack of subplots and that no additional peaks are present after the dark spectrum has been subtracted. This indicates that the MBA is being detected and that there is no significant contamination by other Raman-active molecules in the solution. Figure 2 shows the averaged, baseline-corrected spectra (the final sub-step) at each step of the fabrication process. In Fig. 2, the characteristic MBA peaks at all steps of the fabrication are visible and, in steps 3 to 10 [Figs. 2(c)–2(j)], the pH-sensitive peak at $1430\;{\mathrm{cm}}^{-1}$ is visible. The appearance of a peak at $1430\;{\mathrm{cm}}^{-1}$ at step 3 [Fig. 2(c)] was initially attributed to ethanol, but this was ruled out through parallel studies (Figs. S11 and S12 in the Supplementary Material). Alternatively, the appearance of the peak could be the result of the calcium ions introduced in step 3; this remains under investigation but is not considered to have any bearing on the current studies.

### 3.2 Static Measurements

**Fig. 3:**
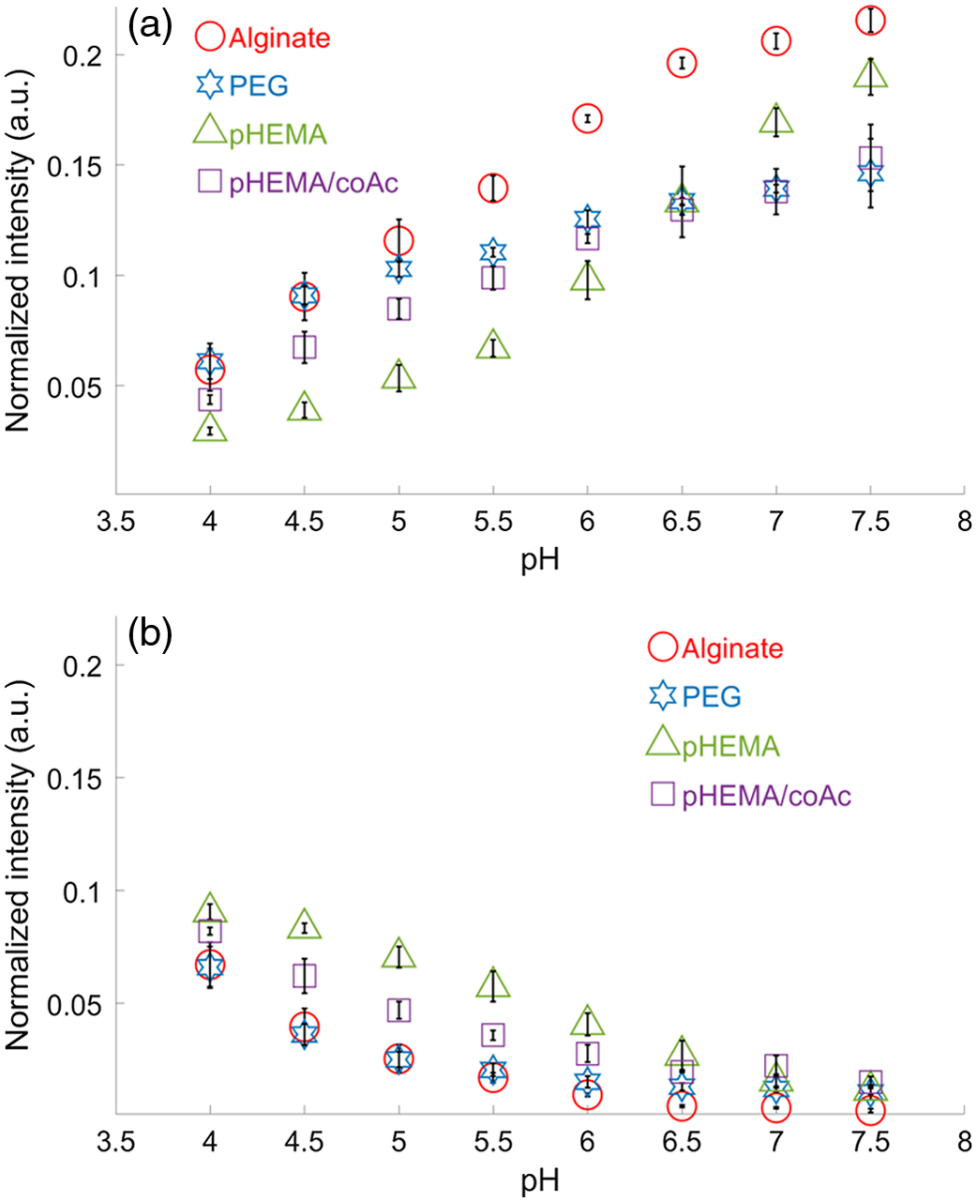
Raman pH sensitive peak intensity versus static pH level of five measurements of five punches of the four gel types. (a) The $1430\text{-}{\mathrm{cm}}^{-1}$ normalized peak average with standard deviation error bars. (b) The $1702\text{-}{\mathrm{cm}}^{-1}$ normalized peak average with standard deviation error bars.

Following the method described in Sec. 2.3.5, the results of static pH sensing are summarized in Fig. 3. This figure shows the two pH-sensitive peak intensities in arbitrary units (a.u.) at Raman shifts of 1430 and $1702\;{\mathrm{cm}}^{-1}$. These intensities have been normalized by the $1582\text{-}{\mathrm{cm}}^{-1}$ peak and averaged over five punches of each gel type. The same intensity scale (y axis) is used for both Figs. 3(a) and 3(b) to clearly show how much more variation there is in the $1430\text{-}{\mathrm{cm}}^{-1}$ peak than the $1702\text{-}{\mathrm{cm}}^{-1}$ peak. These values and the corresponding pH sensitivity values—calculated as the total and percentage change in normalized intensity over the entire pH range tested—are tabulated in Table 1.

**Table 1:** Range of 1430- and $1702\text{-}{\mathrm{cm}}^{-1}$ normalized peak averages of all punches (N=5) of all four gel types.

Gel type	Peak (${\mathrm{cm}}^{-1}$)	Minimum normalized intensity (a.u.)	Maximum normalized intensity (a.u.)	Range = maximum − minimum (a.u.)	Range/minimum (%)
Alginate	1430	0.057	0.216	0.158	275.525
	1702	0.003	0.067	0.065	2521.749
PEG	1430	0.061	0.146	0.085	139.083
	1702	0.011	0.066	0.056	524.747
pHEMA	1430	0.030	0.190	0.160	540.400
	1702	0.012	0.091	0.079	685.512
pHEMA-coA	1430	0.044	0.153	0.109	249.385
	1702	0.015	0.082	0.067	443.225

The dominant result of Fig. 3 and Table 1 is that all four gel types show a positive correlation of normalized intensity with pH for the $1430\text{-}{\mathrm{cm}}^{-1}$ peak and a negative correlation of normalized intensity with pH for the $1702\text{-}{\mathrm{cm}}^{-1}$ peak. The range of $1430\text{-}{\mathrm{cm}}^{-1}$ peak values is much greater in magnitude (more than 2×) than the range of the $1702\text{-}{\mathrm{cm}}^{-1}$ peak values. These observations suggest that none of the gels substantially inhibited the $H^{+}$ ions in the buffer from interacting with the MBA inside the MCs. It also means that none of the gels contaminated the MBA’s Raman signal by introducing their own intrinsic Raman signals.

More specific results of Fig. 3 and Table 1 are that alginate and pHEMA have a larger dynamic range (∼0.16  a.u.) than the other two gels (<0.1  a.u.) for the $1430\text{-}{\mathrm{cm}}^{-1}$ peak. It is also interesting to compare the shapes of the alginate and pHEMA curves in Fig. 3. They both exhibit a quasi-sigmoidal shape but are shifted relative to one another. Alginate is more sensitive at low pH levels (up to pH 6.5), whereas pHEMA is more sensitive (indicated by higher slope) at greater pH levels (pH 5.5 and above). For the $1702\text{-}{\mathrm{cm}}^{-1}$ peak, all four gels are much closer in range (0.056 to 0.079 a.u.). Since the $1702\text{-}{\mathrm{cm}}^{-1}$ peak minimum values are so close to zero, they are more affected by signal noise and less reliable than the $1430\text{-}{\mathrm{cm}}^{-1}$ peak values for calibrating to a corresponding pH level.

#### 3.2.1 Reference peaks as pH indicators

Although the 1072- and $1582\text{-}{\mathrm{cm}}^{-1}$ MBA peaks are normally considered to be largely insensitive to pH, some others have reported the lateral shift of these two peaks as a pH indicator.^47^^,^^52^ Here, the intensity of 1072- and $1582\text{-}{\mathrm{cm}}^{-1}$ reference peaks were specifically investigated for pH-sensitivity, using the same approach as the pH-sensitive peaks. As this was not the main focus of this work, details are provided only in the Supplementary Material. It is notable that the range of normalized intensities over the pH range of 4.0 to 7.5 was similar to the magnitude found using the pH-sensitive peaks for the $1072\text{-}{\mathrm{cm}}^{-1}$ peak and was much higher in absolute magnitude (about 5 times). This suggests that using the $1072\text{-}{\mathrm{cm}}^{-1}$ peak to determine pH level shows promise, especially when signal intensities are low and/or noise is high, and this may be a topic of future investigation.

#### 3.2.2 Longevity measurements

**Fig. 4:**
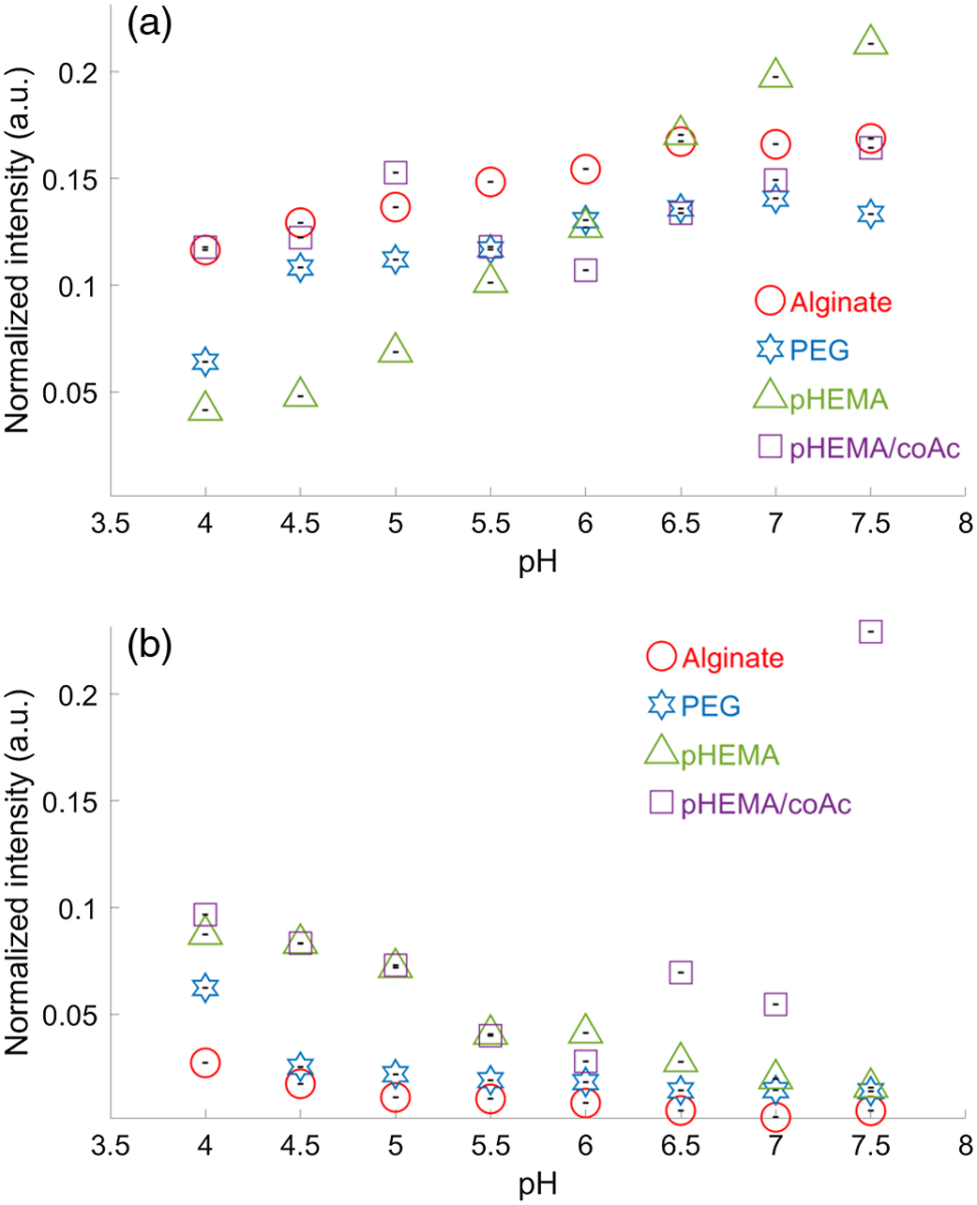
Raman pH-sensitive peak intensity versus static pH level of five measurements of one punch of the four gel types at age five months. (a) The $1430\text{-}{\mathrm{cm}}^{-1}$ normalized peak average with standard deviation error bars. (b) The ${1702\text{-}\mathrm{cm}}^{-1}$ normalized peak average with standard deviation error bars.

The storage stability of the hydrogels was studied by repeating the static measurements (described in Sec. 2.3.5) on a set of aged gels that had been fabricated five months earlier and stored at 4°C. The results, in Fig. 4 and Table 2, can be directly compared with the new gels (Fig. 3 and Table 1). Of the four gel types, pHEMA is the only one that retains (even slightly exceeds) its original dynamic range (0.160 a.u. new versus 0.172 a.u. after 5 months) for the $1430\text{-}{\mathrm{cm}}^{-1}$ normalized peak. The other three gels lost approximately two thirds of their range. For example, alginate changed from 0.158 a.u. (new) to 0.052 a.u. (aged) for the $1430\text{-}{\mathrm{cm}}^{-1}$ normalized peak. In one instance, outliers that did not follow the expected trends observed in other materials were seen in the measurements for pHEMA-coA at pH 6.5 to 7.5 for the $1702\;{\mathrm{cm}}^{-1}$ normalized peak; in particular, the value of >0.2 at pH 7.5 exceeds all other documented values. This behavior was only noted in the aged samples, and may be indicative of some degree of gel degradation during extended storage. The loss in the dynamic range of the sensors embedded in all of the materials except pHEMA indicates than those materials (alginate, PEG, and pHEMA-coA) could be either partially disintegrating, thereby allowing the sensors to escape, or the matrix structure could be collapsing and thereby reducing permeability and porosity. Since only a single punch was available for each gel at the five months mark (five punches were used for the initial static measurements in Sec. 3.2), the aged dataset could be expanded in future study. The reference peaks of the aged gels could be used as alternative pH-sensors (as proposed in Sec. 3.2.1) because of their much higher peak intensity magnitudes and, in the case where the gels are breaking down, they may have more robust signals at these peaks in the aged gels.

**Table 2:** Range of 1430- and ${1702\text{-}\mathrm{cm}}^{-1}$ normalized peak averages of one punch of gels at age 5 months.

Gel type	Peak (${\mathrm{cm}}^{-1}$)	Minimum normalized intensity (a.u.)	Maximum normalized intensity (a.u.)	Range = maximum − minimum (a.u.)	Range/minimum (%)
Alginate	1430	0.116	0.169	0.052	44.960
	1702	0.001	0.027	0.026	1726.356
PEG	1430	0.064	0.140	0.077	119.618
	1702	0.014	0.062	0.048	354.527
pHEMA	1430	0.041	0.213	0.172	417.146
	1702	0.015	0.087	0.072	463.718
pHEMA/coAc	1430	0.107	0.164	0.058	53.819
	1702	0.028	0.229	0.201	725.290

### 3.3 Dynamic Measurements

**Fig. 5:**
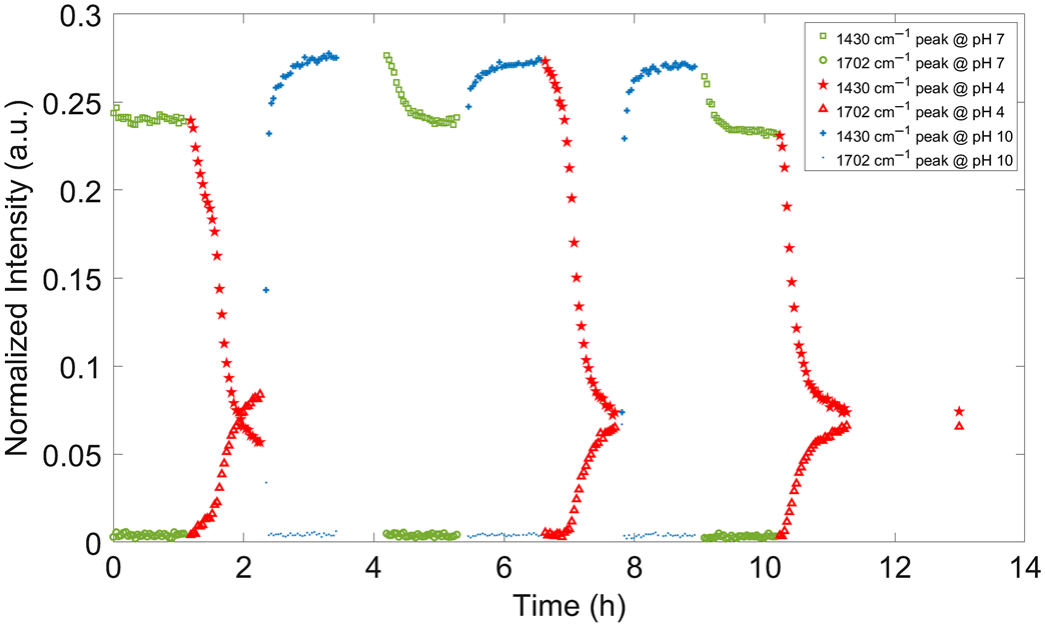
Example flow cell study: the alginate series 1 (as shown in Fig. S14 in the Supplementary Material). At pH 7, the normalized intensities at 1430 and $1702\;{\mathrm{cm}}^{-1}$ are shown using the symbol of a square and a circle, respectively. At pH 4, the normalized intensities at 1430 and $1702\;{\mathrm{cm}}^{-1}$ are shown using the symbol of a star and a triangle, respectively. At pH 10, the normalized intensities at 1430 and $1702\;{\mathrm{cm}}^{-1}$ are shown using the symbol of a plus sign and a period, respectively.

Following the method described in Sec. 2.3.6, the dynamic response of the hydrogels to changes in pH was evaluated. Twelve flow cell studies were completed, three “series” for each of the four gel types. Representative data from one of the studies are shown in Fig. 5, where the normalized intensity at the two pH-sensitive peaks is plotted versus time as the pH level inside the flow cell was changed approximately every 2 h. The complete set of flow cell study plots are provided in Figs. S14-S25 in the Supplementary Material.

The most obvious feature of Fig. 5 is the full reversibility (repeatability) of the sensors, which is revealed when the normalized intensity returns to approximately the same value for each pH level as steady state is approached. As stated at the outset, reversibility is required for a reliable biosensor because it enables calibration of the analyte concentration (pH) to the normalized peak intensity. Another striking feature of Fig. 5 is that the response is most sensitive in the lower pH range (between pH 4 and 7). This matches previous results concluding that MBA is useful as a pH sensor in an acidic environment, but that it is not well-suited as a pH sensor in alkaline environments. This feature is present for both peaks but is even more apparent in the ${1702\text{-}\mathrm{cm}}^{-1}$ peak, which shows no distinguishable difference in intensity between pH 7 and pH 10. This result was also observed in Sec. 3.2 where, by pH 7, the magnitude of the ${1702\text{-}\mathrm{cm}}^{-1}$ peak was already within the spectral noise.

**Fig. 6:**
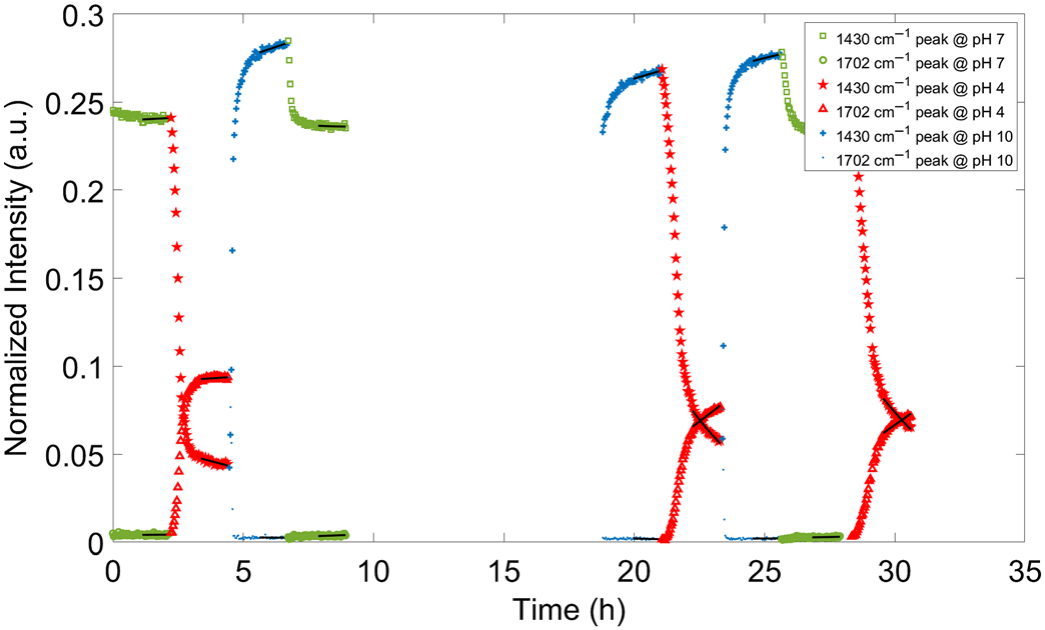
Curve fitting example. Exponential curves are fit to the last part of each pH segment and drawn as solid lines overlaid on the symbols marking the measured intensities. At pH 7, the normalized intensities at 1430 and $1702\;{\mathrm{cm}}^{-1}$ are shown using the symbol of a square and a circle, respectively. At pH 4, the normalized intensities at 1430 and $1702\;{\mathrm{cm}}^{-1}$ are shown using the symbol of a star and a triangle, respectively. At pH 10, the normalized intensities at 1430 and $1702\;{\mathrm{cm}}^{-1}$ are shown using the symbol of a plus sign and a period, respectively.

To evaluate the response time of the gels, a novel data analysis technique was required as this is the first known study attempting to quantify the speed of response to changing pH in real time. Instantaneous slope just after the buffer was changed to a new pH level provides one measure of speed of response. However, a more reliable method was identified using exponential curve-fitting of the end of a pH segment to determine a measure of steady-state and the determination of the time constant, τ, associated with the rate of response. Exponential curve fitting was applied to the normalized intensity curves of the two pH-sensitive peaks versus time to estimate time constants for each of the nine pH segments, for each of the three series and for each of the four gel types (total: 2×9×3×4=216 time constants). The curve fitting used one of the exponential equations:

$$y=ae^{-bx}+c$$ (1)

or

$$y=a(1-e^{-bx})+c,$$ (2)

based on the direction of approach toward steady state. Equation (1) was used when steady state was approached by decay from higher values and Eq. (2) was used when steady state was approached from lower values. The time constant, τ, is equivalent to −1/b, in both equations. At x=τ, the exponential term becomes $e^{-1}$, which evaluates to 0.368 (or 36.8%) and maps to 62.2% (i.e., 100% to 36.8%) of the final steady-state value. MATLAB^®^ 2019 curve fitting function was used for this analysis. The results of exponential curve fitting are overlaid in black over the last 29 points (right side) of each pH segment shown in Fig. 6.

**Fig. 7:**
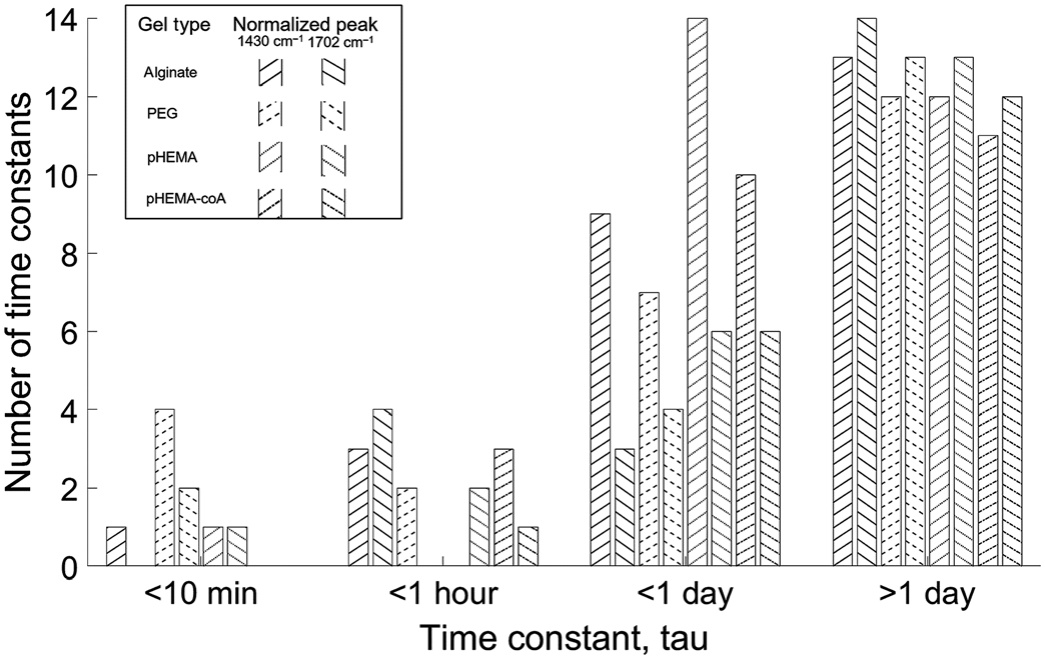
Distribution of time constants for the normalized intensities of two pH-sensitive peaks, at 1430 and $1702\;{\mathrm{cm}}^{-1}$, for all pH segments, all series, and all gel types (alginate, PEG, pHEMA, and pHEMA-coA). Eight different fill patterns are defined in the legend to represent the eight cases.

The results of the curve-fitting for all 12 flow cell studies are combined in Fig. 7, which displays the time constants determined for all gels, all series, and all pH segments and sorted into bins by magnitude. A log scale is used to accommodate the large range of τ values and to display the smallest τ values in higher resolution since these represent the gels that are potentially the most useful as real-time *in vivo* sensors.

In Fig. 7, the gel with the greatest number of time constants in the interval between 0 and 10 min is PEG, with a total of four $1430\text{-}{\mathrm{cm}}^{-1}$ peak values and two $1702\text{-}{\mathrm{cm}}^{-1}$ peak values. In contrast, pHEMA only had two cases with such rapid response, alginate had only one, and pHEMA-coA had none. Since the bulk of the cases show time constants in the intervals: >1  h and >1  day, it must be concluded that these materials are not viable for real-time tracking of *in vivo* metabolites in many practical situations where rapid fluctuations may be expected. However, many metabolites do not change so quickly and thus these materials could be suitable for periodic sampling. Additional plots showing how the time constants are distributed across pH levels are also included in Figs. S39-S41 in the Supplementary Material. The most significant observation in those plots is that PEG is present in the 10 min or less interval for all three pH levels, whereas alginate is only present at pH 7, and pHEMA is present at both pH 7 and pH 10.

### 3.4 Consistency of Static and Dynamic Measurements

**Fig. 8:**
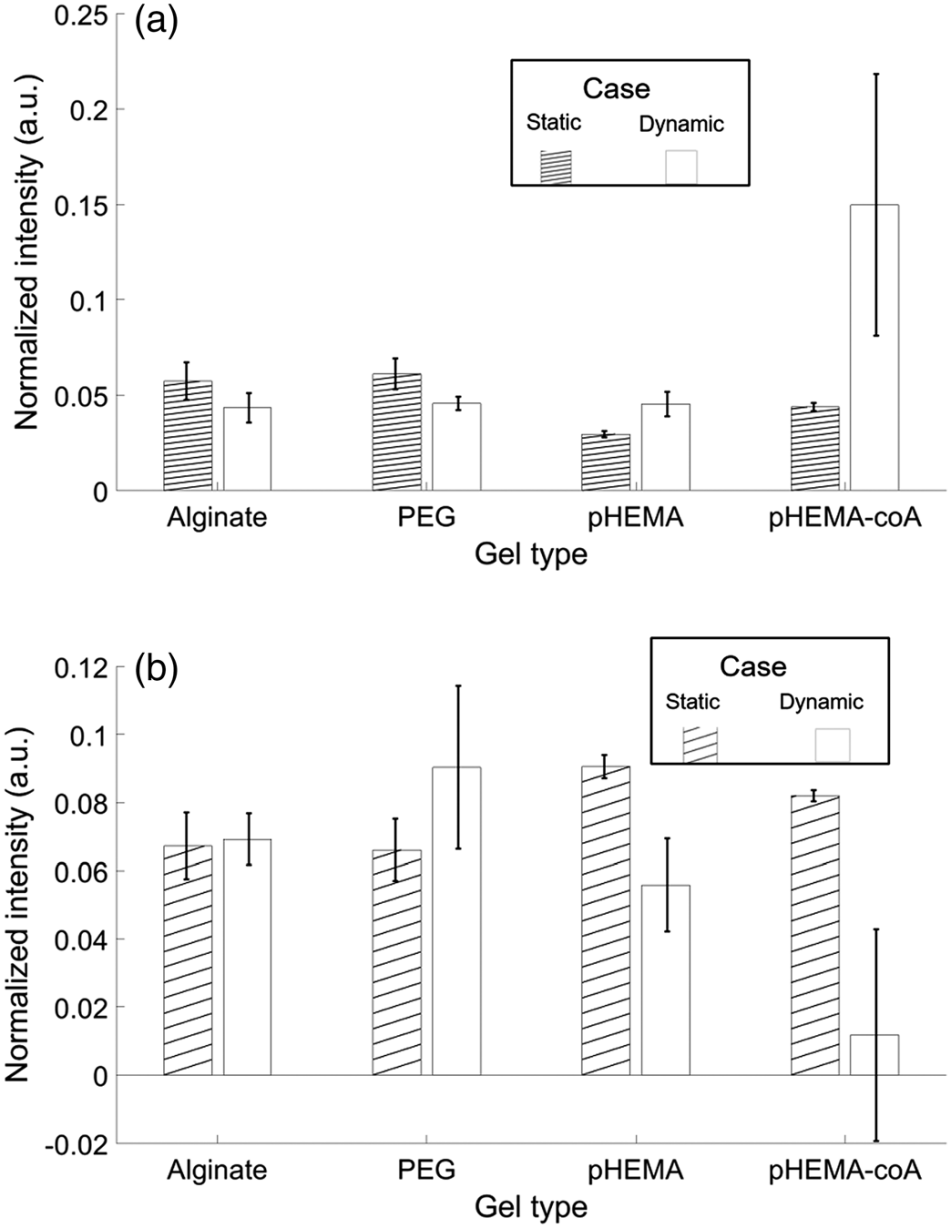
Consistency of hydrogels under static (hatched bar) versus dynamic (plain bar) conditions at pH 4. Average over all measurements with error bars indicating ±1 standard deviation from the average (a) $1430\text{-}{\mathrm{cm}}^{-1}$ normalized peak and (b) $1702\text{-}{\mathrm{cm}}^{-1}$ normalized peak. Note that vertical scales differ between (a) and (b).

**Fig. 9:**
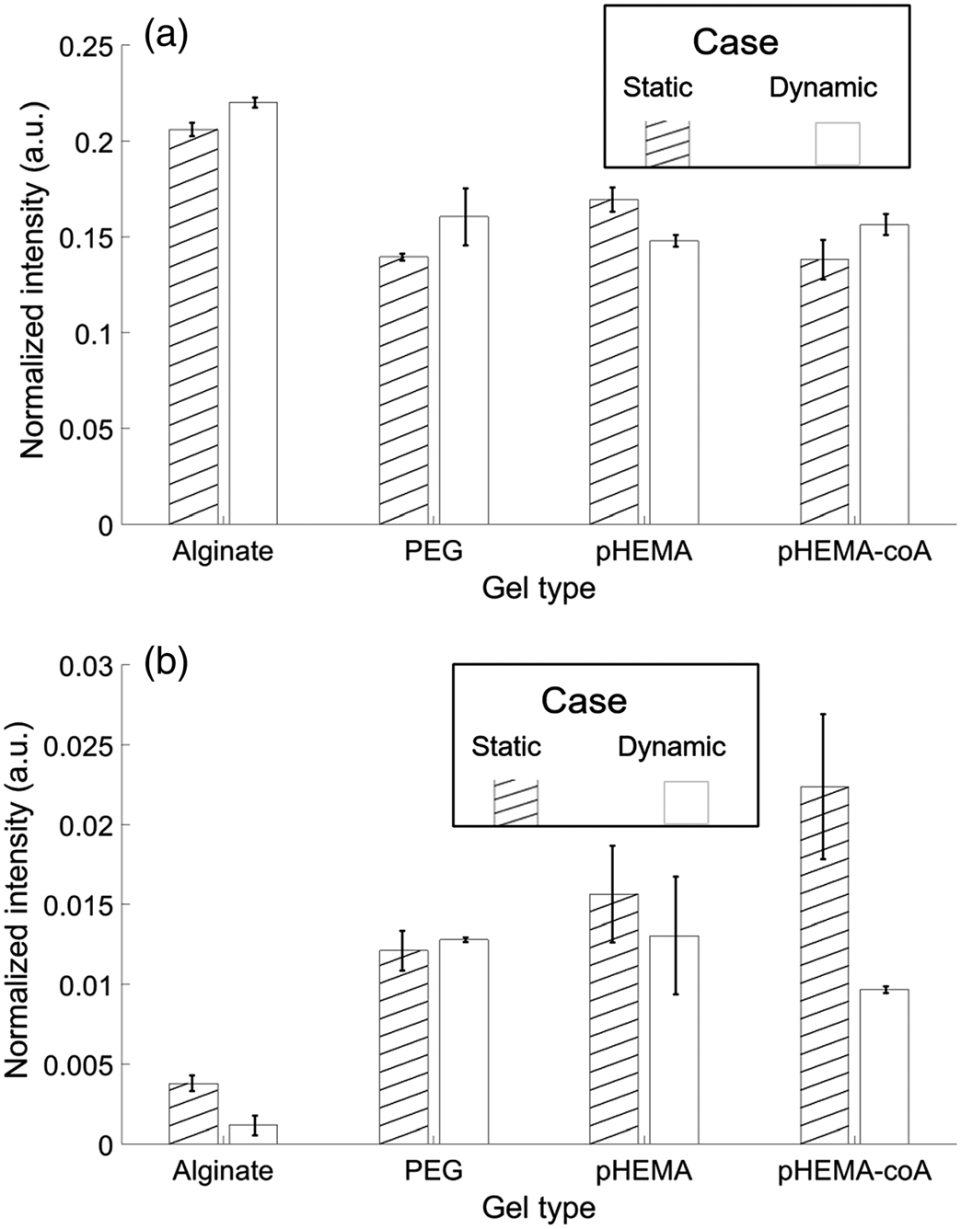
Consistency of hydrogels under static (hatched bar) versus dynamic (plain bar) at pH 7. Average over all measurements with error bars indicating ±1 standard deviation from the average (a) $1430\text{-}{\mathrm{cm}}^{-1}$ normalized peak and (b) $1702\text{-}{\mathrm{cm}}^{-1}$ normalized peak. Note that vertical scales differ between (a) and (b).

This section compares the static and dynamic measurements for consistency at common pH levels. The premise is that, if the sensors in the hydrogels under dynamic pH have achieved steady-state, then the pH-sensitive peak intensities should match those of the sensors in the hydrogels under static pH. Since the static measurements were collected in environments of pH 4 through pH 7.5, in steps of 0.5 and since the dynamic measurements were collected in environments of pH 4, 7, and 10, the two overlapping cases to compare are pH 4 (Fig. 8) and pH 7 (Fig. 9). On the left of each pair of bars in Figs. 8 and 9, the hatched bar is the average of the normalized peak over all punches (N=5) in static conditions. On the right of each pair of bars, the plain bar is the average of the normalized peak over all the series and three pH segments (of each pH level) in dynamic conditions.

Figures 8 and 9 display the error bars calculated from the standard deviation of the measurements averaged over all punches (N=5) for the hatched bars, and as the standard deviation of the measurements averaged over 3 series and 3 segments (N=9) for the plain bars. The Supplementary Material shows how the magnitude of the error bars is affected when the original raw spectra are used instead of the averages. Using the raw spectra changes N from 5 to 125 for the static case and changes N from 9 to 45 for the dynamic case (Fig. S42 in the Supplementary Material).

Figure 8 shows that, at pH 4, the alginate, PEG, and pHEMA values agree within 20% across static and dynamic conditions (although not within the error bars, in the pHEMA case), whereas the pHEMA-coA values diverge greatly [by more than 100% in Fig. 8(a)] and are outside of the error bars [in both Figs. 8(a) and 8(b)]. The dynamic measurement values of pHEMA-coA in both Figs. 8(a) and 8(b) suggest that with additional time, they could converge on the static measurements. This is because pH 4 is the lowest pH level (corresponding to lowest $1430\text{-}{\mathrm{cm}}^{-1}$ peak and highest $1702\text{-}{\mathrm{cm}}^{-1}$ peak) and means that steady-state is being approached from above. The standard deviation error bars in Fig. 8 show that all but the dynamic measurements of pHEMA-coA have low variance (5% to 10% of average). The high variance of the dynamic measurements of the pHEMA-coA shows poor consistency (even across dynamic measurements) and therefore less useful in a dynamic environment.

In Fig. 9, the pH 7 case shows improved consistency in all of the gels (compared to pH 4 in Fig. 8) as the static and dynamic measurements agree to within 10%. There is also a reduction in variance in all of the measurements. As noted earlier, the fact that MBA’s response is more dynamic in acidic environments helps to the reduced variance in the dynamic measurements here since when the pH is changing from 10 to 7, there is less change in the peaks (than when pH is changed from 10 to 4 or from 7 to 4).

## 4 Conclusions

This work evaluated the performance of SERS-based pH sensors using microencapsulated AuNPs embedded in different hydrogels. These systems present solutions for a pseudo-solid-state pH sensing approach with the potential for continuous monitoring, with consideration that different hydrogels may affect the sensing performance in terms of sensitivity (dynamic range), longevity (dynamic range over time), reversibility (repeatability), responsivity (to a changing environment), and consistency (matching static and dynamic measurements). It was determined that Raman spectra from all four hydrogel types (alginate, PEG, pHEMA, and pHEMA-coA) exhibited sensitivity to pH from 4 to 7.5 in static conditions as well as dynamically changing pH environments. Further, all four types of hydrogel showed full reversibility after step changes in pH; Raman signals measured in dynamic (flow) conditions were found to be within 10% to 20% of the levels measured in static conditions for pH 4 and 7. Further, aged gels tested after five months of storage still responded to pH challenges, although most formulations lost more than half of the dynamic response over this time. It was also noted that the time required to reach true steady state was relatively long (hours to days). Taken together, these observations suggest that sensing systems based on these materials have potential for long-term monitoring where analyte levels are not expected to fluctuate rapidly.
