# Supplementary material for: Comparison of SERS pH probe responses after microencapsulation within hydrogel matrices
Source: J Biomed Opt. 2021 Sep 13;26(9):097001. doi: 10.1117/1.JBO.26.9.097001 (PMC8435981; doi:10.1117/1.JBO.26.9.097001)
Supplement: Supplementary file 1 [file JBO_026_097001_SD001.pdf]

# Supplementary Material for
# Comparison of SERS pH Probe Responses after Microencapsulation within Biocompatible Hydrogel Matrices

**Dayle Kotturi,[a] Sureyya Paterson,[a] Mike McShane[a,b,*]**
[a] Department of Biomedical Engineering, Texas A&M University, College Station, TX USA 77843;
[b] Department of Materials Science and Engineering, Texas A&M University, College Station, TX, USA 77843

***** e-mail: mcshane@tamu.edu; phone 1 979 845-7941; fax 1 979 845-4450; biosym.org

## 1 Verification of sensing material

The figures S1–S10 show the spectra of the SERS active sensing materials at ten steps in the manufacturing process. Each figure displays a series of spectra starting with the "dark" at the top. The "dark" shows the spectrum of the background of the room (laser off). Below that is one sample of the five raw spectra of the AuNPs with MBA (laser on). The laser power and integration time were selected such that the intensity of the raw MBA peaks (at 1072 $cm^{-1}$ and 1582 $cm^{-1}$) were at least an order of magnitude more than the signal noise. In this case, the raw peak intensities are ~1600 arbitrary units (a.u.) and the signal noise has a magnitude of ~100 a.u. The raw spectrum with the dark subtracted is next and is followed by the average of the five raw spectra, after dark subtraction. The baseline of the averaged spectrum is shown next, followed by the average spectrum with the baseline removed. The final plot in the stack shows the normalized, baseline-corrected average spectrum. The normalization uses the average of 5 values centered at 1582 $cm^{-1}$ reference peak (rather than a single intensity). The purpose of figures S1-S10 is to demonstrate that the standard MBA peaks are present, that the standard peaks are at least an order of magnitude greater than the noise and that no contamination is present in the form of unexpected peaks. Figures S11 and S12 show the effect of introducing ethanol to the system, in order to examine its effect on the 1430 $cm^{-1}$ peak. Although ethanol's presence did not seem to cause the 1430 $cm^{-1}$ peak to appear (as expected), this has no effect on the overall results of the study.

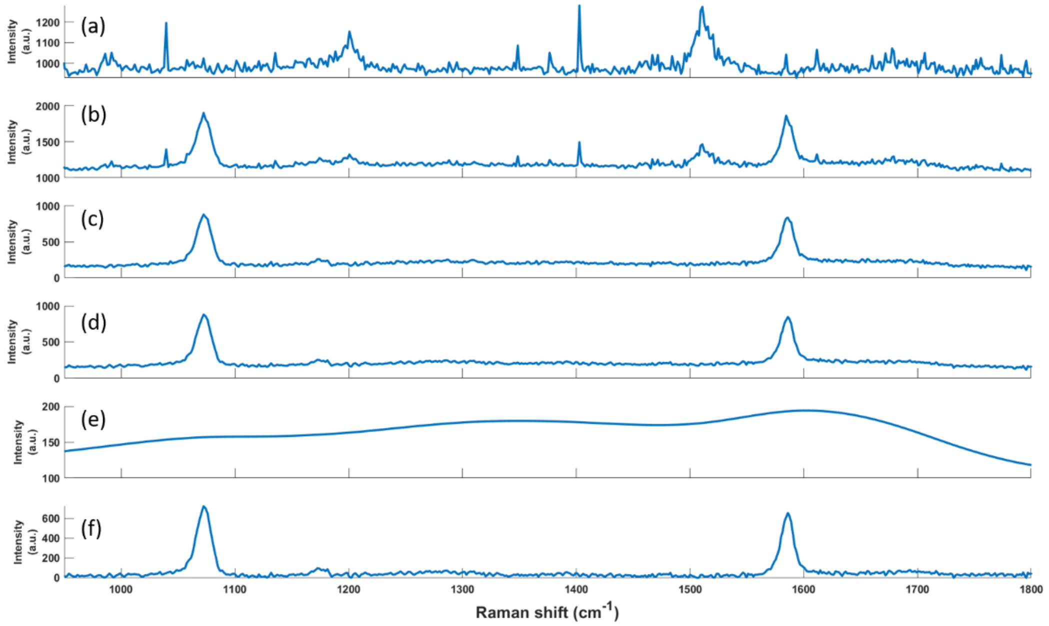


**Fig. S1** Step 1 of the fabrication process: attaching the MBA to the AuNPs. a) dark spectrum (laser is off) b) raw spectrum (laser is on) c) raw – dark d) average of 5 successive raw spectra with dark subtracted e) baseline correction determined from asymmetric least squares f) average – baseline correction.

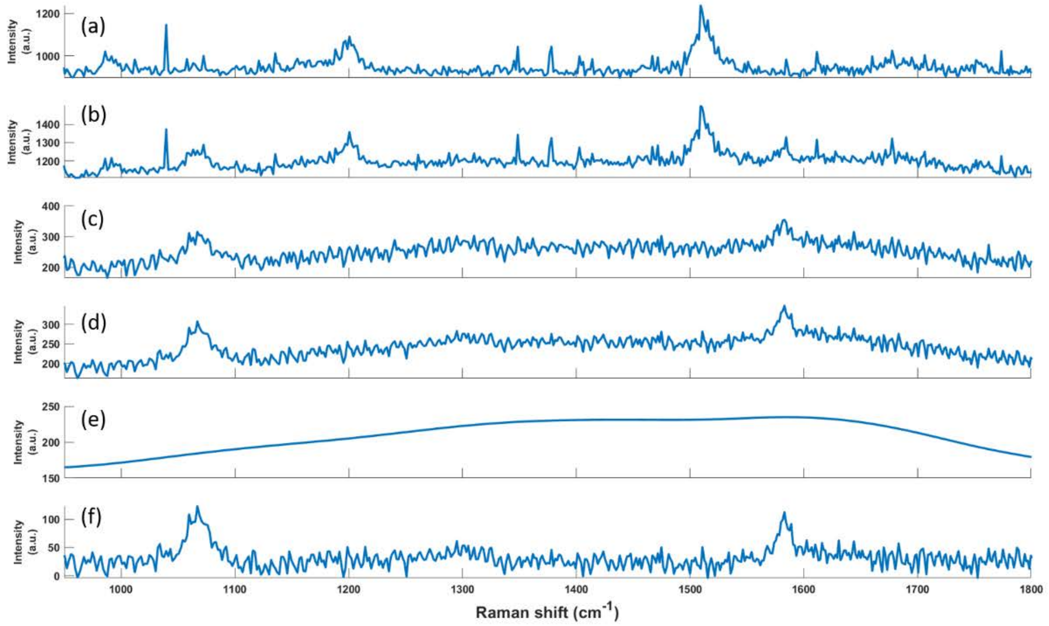


**Fig. S2** Step 2 of the fabrication process: MBA AuNPs into $NaCO_3$. a) dark spectrum (laser is off) b) raw spectrum (laser is on) c) raw – dark d) average of 5 successive raw spectra with dark subtracted e) baseline correction determined from asymmetric least squares f) average – baseline correction.

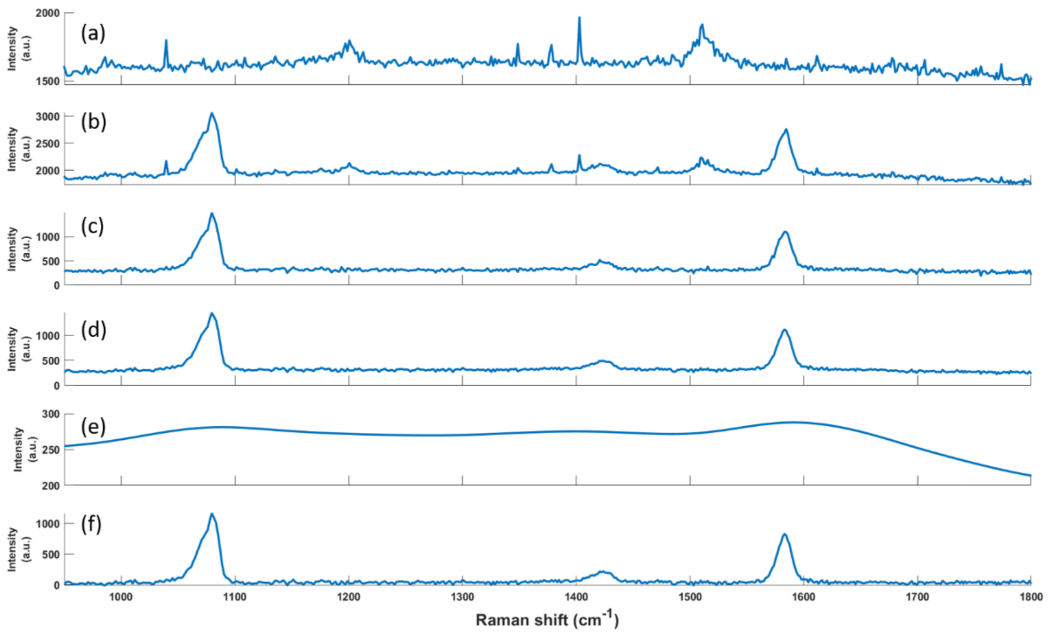


**Fig. S3** Step 3 of the fabrication process: MBA AuNPs with $NaCO_3$ and $CaCl_2$. a) dark spectrum (laser is off) b) raw spectrum (laser is on) c) raw – dark d) average of 5 successive raw spectra with dark subtracted e) baseline correction determined from asymmetric least squares f) average – baseline correction.

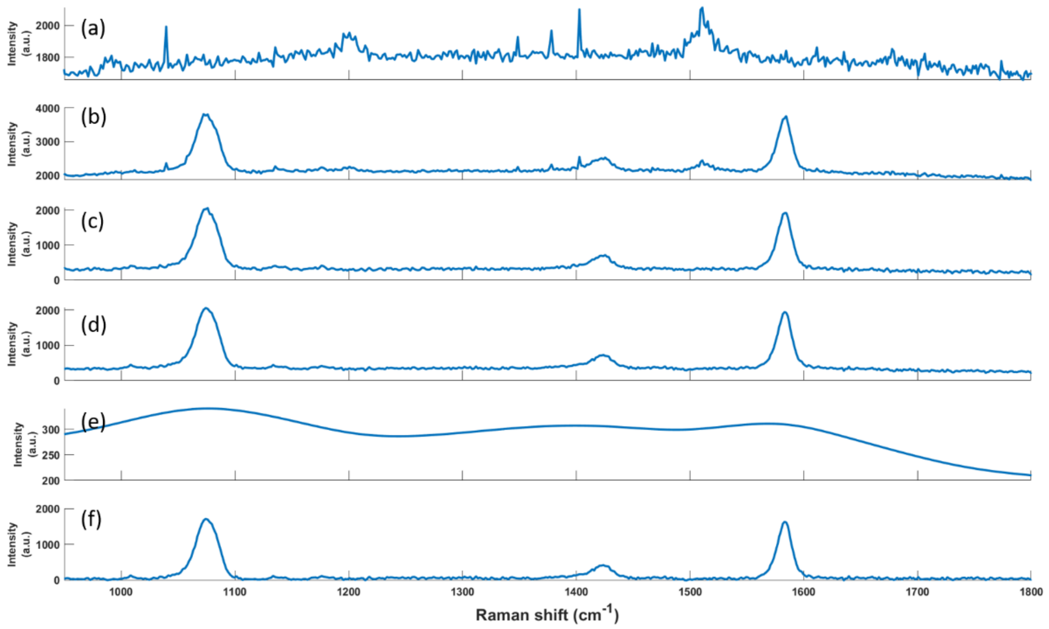

**Fig. S4** Step 4 of the fabrication process: first wash with $NaCO_3$. a) dark spectrum (laser is off) b) raw spectrum (laser is on) c) raw – dark d) average of 5 successive raw spectra with dark subtracted e) baseline correction determined from asymmetric least squares f) average – baseline correction.

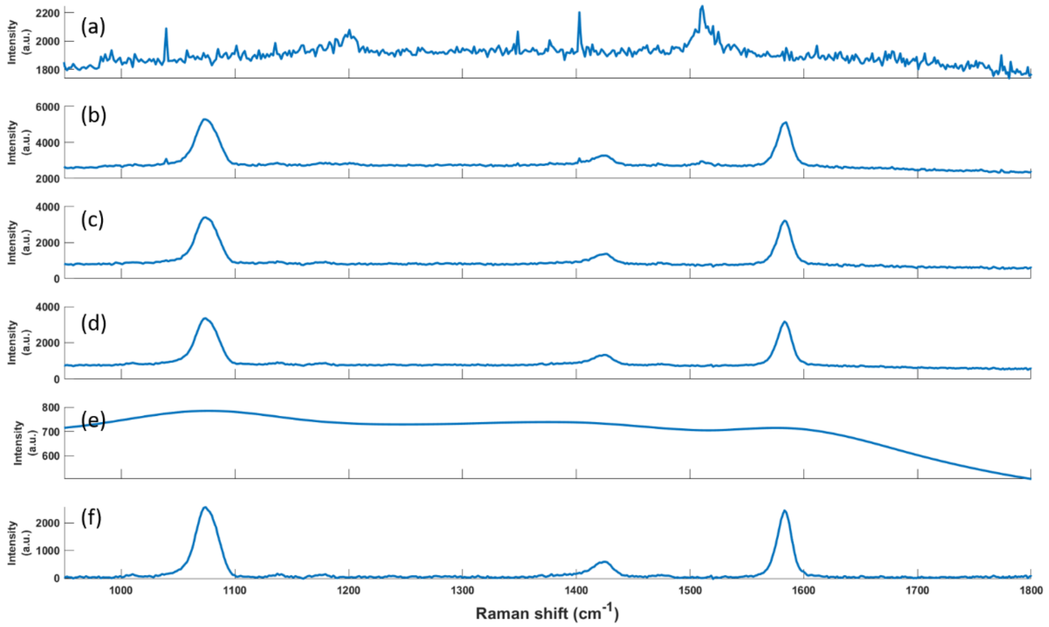


**Fig. S5** Step 5 of the fabrication process: after first bilayer PDADMAC/PSS. a) dark spectrum (laser is off) b) raw spectrum (laser is on) c) raw – dark d) average of 5 successive raw spectra with dark subtracted e) baseline correction determined from asymmetric least squares f) average – baseline correction.

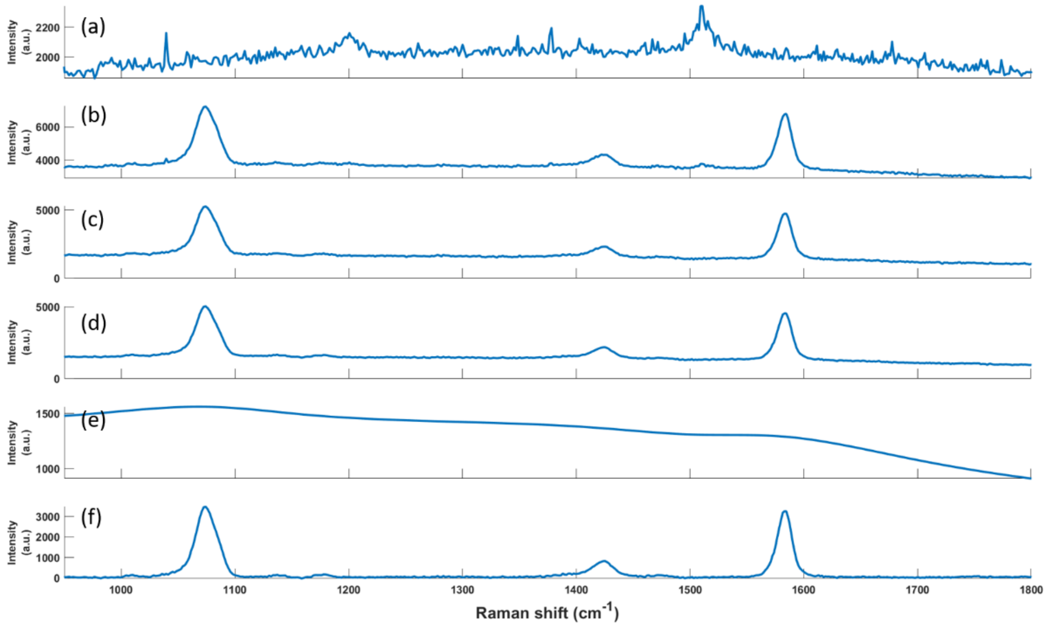

**Fig. S6** Step 6 of the fabrication process: after five bilayers. a) dark spectrum (laser is off) b) raw spectrum (laser is on) c) raw – dark d) average of 5 successive raw spectra with dark subtracted e) baseline correction determined from asymmetric least squares f) average – baseline correction.

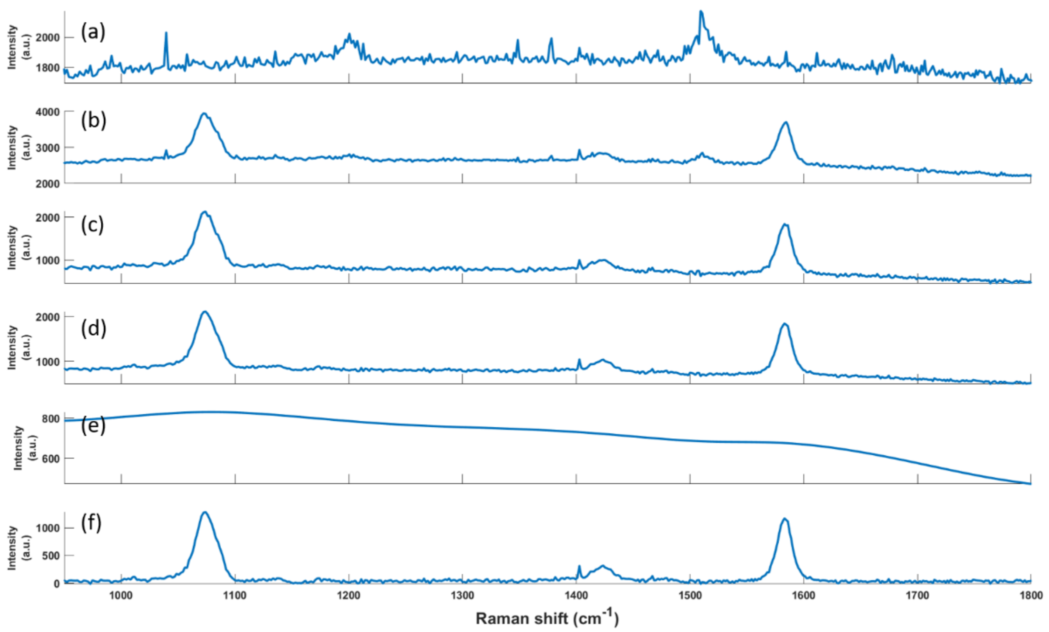


**Fig. S7** Step 7 of the fabrication process: after ten bilayers. a) dark spectrum (laser is off) b) raw spectrum (laser is on) c) raw – dark d) average of 5 successive raw spectra with dark subtracted e) baseline correction determined from asymmetric least squares f) average – baseline correction.

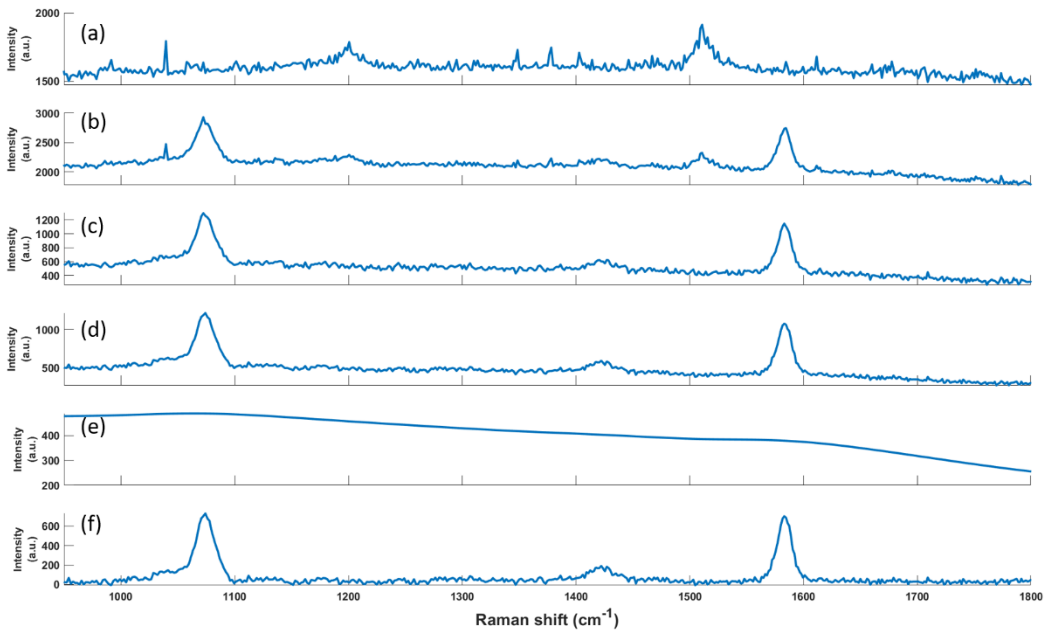


**Fig. S8** Step 8 of the fabrication process: after first wash. a) dark spectrum (laser is off) b) raw spectrum (laser is on) c) raw – dark d) average of 5 successive raw spectra with dark subtracted e) baseline correction determined from asymmetric least squares f) average – baseline correction.

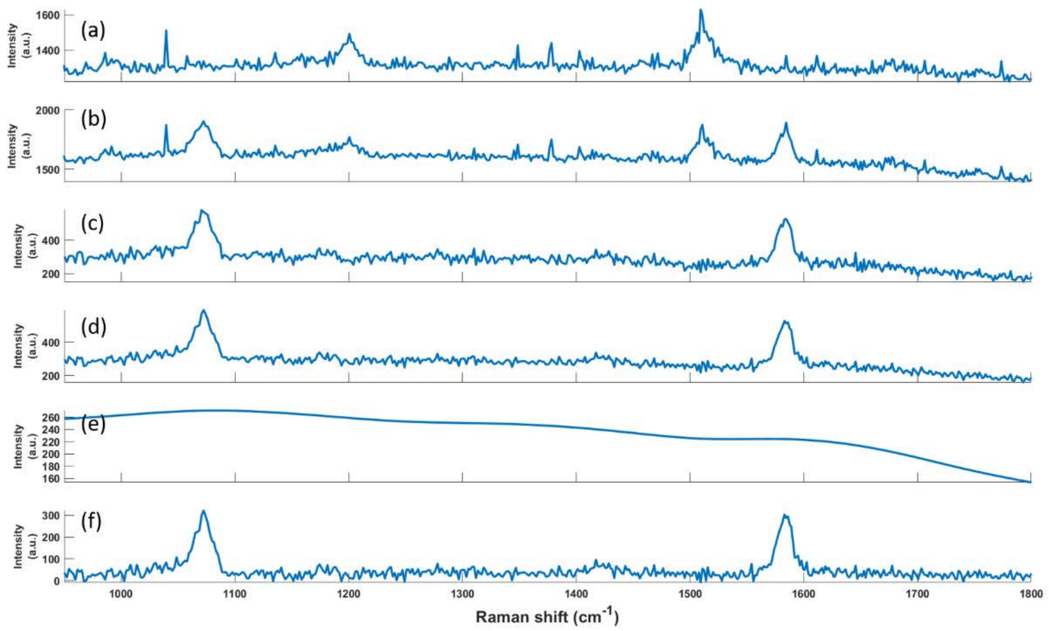


**Fig. S9** Step 9 of the fabrication process: after third wash and into 10mM pH7. a) dark spectrum (laser is off) b) raw spectrum (laser is on) c) raw – dark d) average of 5 successive raw spectra with dark subtracted e) baseline correction determined from asymmetric least squares f) average – baseline correction.

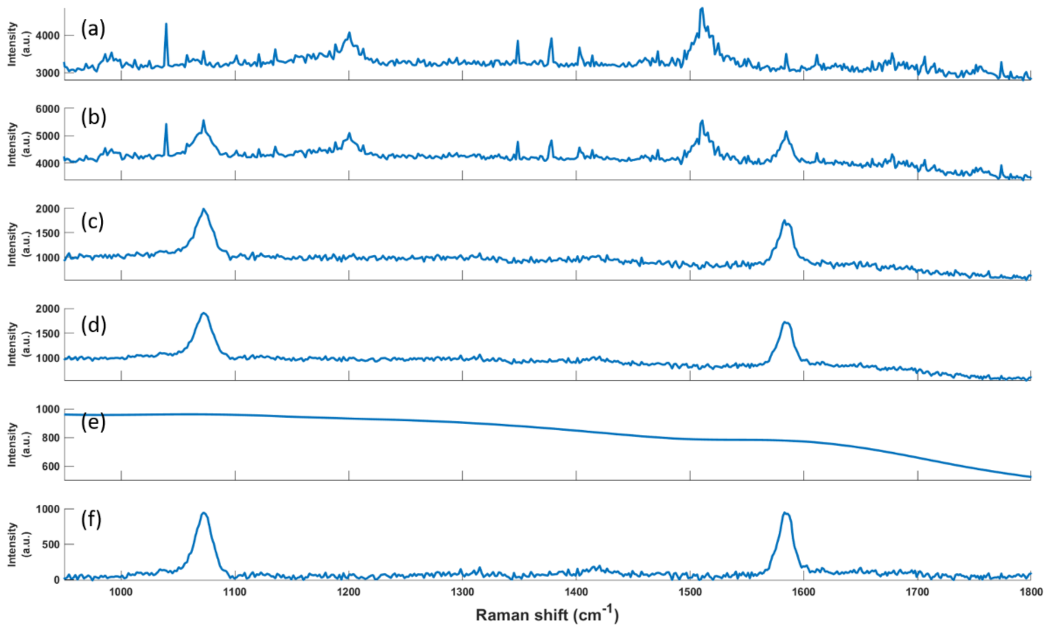


**Fig. S10** Step 10 of the fabrication process: in alginate gel at pH7. a) dark spectrum (laser is off) b) raw spectrum (laser is on) c) raw – dark d) average of 5 successive raw spectra with dark subtracted e) baseline correction determined from asymmetric least squares f) average – baseline correction.

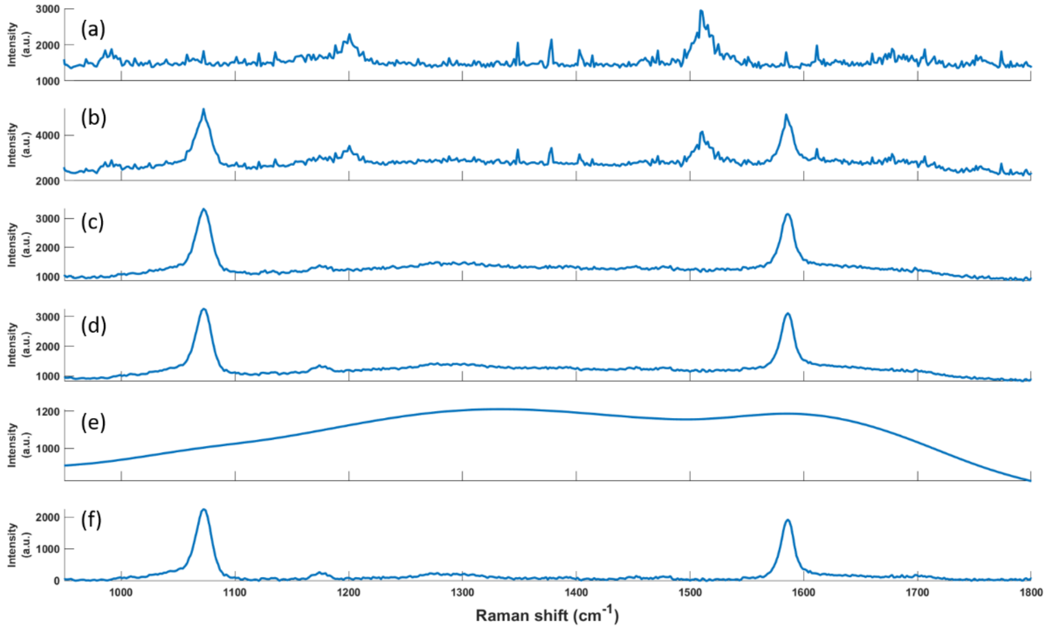


**Fig. S11** Checking the effect of MBA pH-sensitive peak formation: without ethanol. a) dark spectrum (laser is off) b) raw spectrum (laser is on) c) raw – dark d) average of 5 successive raw spectra with dark subtracted e) baseline correction determined from asymmetric least squares f) average – baseline correction.

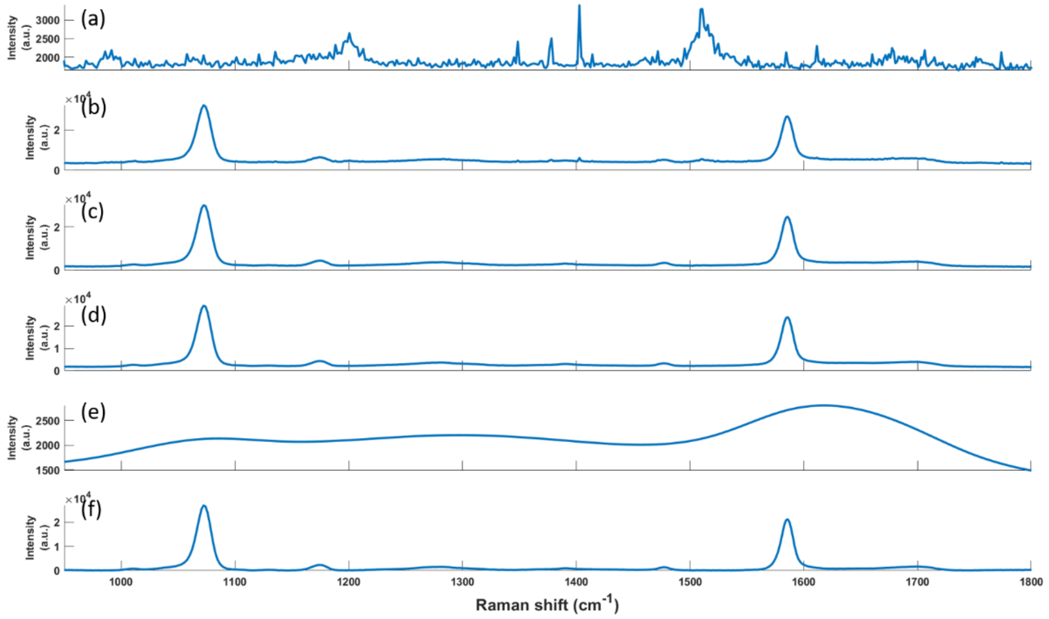


**Fig. S12** Checking the effect of MBA pH-sensitive peak formation: with ethanol. a) dark spectrum (laser is off) b) raw spectrum (laser is on) c) raw – dark d) average of 5 successive raw spectra with dark subtracted e) baseline correction determined from asymmetric least squares f) average – baseline correction.

## 2 Static measurements

### *2.1 Reference peaks*

Although not the focus of the work, it may be useful to look at other options for measuring pH for cases when the Raman intensities are very weak and/or when the signal noise is high. Under these circumstances, the primary pH-sensitive peaks may be indistinguishable from zero, making them ineffective as pH indicators. In their place, the stronger reference peaks could possibly be used. It is also possible that the pH-sensitivity of the reference peaks could be used in conjunction with the pH-sensitive peaks to improve the estimation of pH using multiple inputs.

Although the 1072 $cm^{-1}$ and 1582 $cm^{-1}$ MBA peaks are normally considered insensitive to pH, some pH-sensitivity was observed in this study. The changes in magnitudes of 1072 $cm^{-1}$ and 1582 $cm^{-1}$ reference peaks were investigated for pH-sensitivity. Raman intensities at the 1072 $cm^{-1}$ and

1582 $cm^{-1}$ reference peaks were normalized (by each other) and plotted vs pH in Fig. S13a and 13b, respectively, with values tabulated in Table S1. The dynamic range of normalized intensities corresponding to pH changing from 4.0 to 7.5, was similar to the magnitude found using the pH-sensitive peaks (0.16 a.u.) for the 1072 $cm^{-1}$ peak; it was lower for the 1582 $cm^{-1}$ (0.14 a.u.). The gels with the largest range were pHEMA and pHEMA-coA. The fact that the range of the 1072 $cm^{-1}$ peak matched the range of the 1430 $cm^{-1}$ peak but was much higher in absolute magnitude (about 5 times), indicates that using the 1072 $cm^{-1}$ peak to determine pH level shows promise, especially when signal intensities are low and/or noise is high.

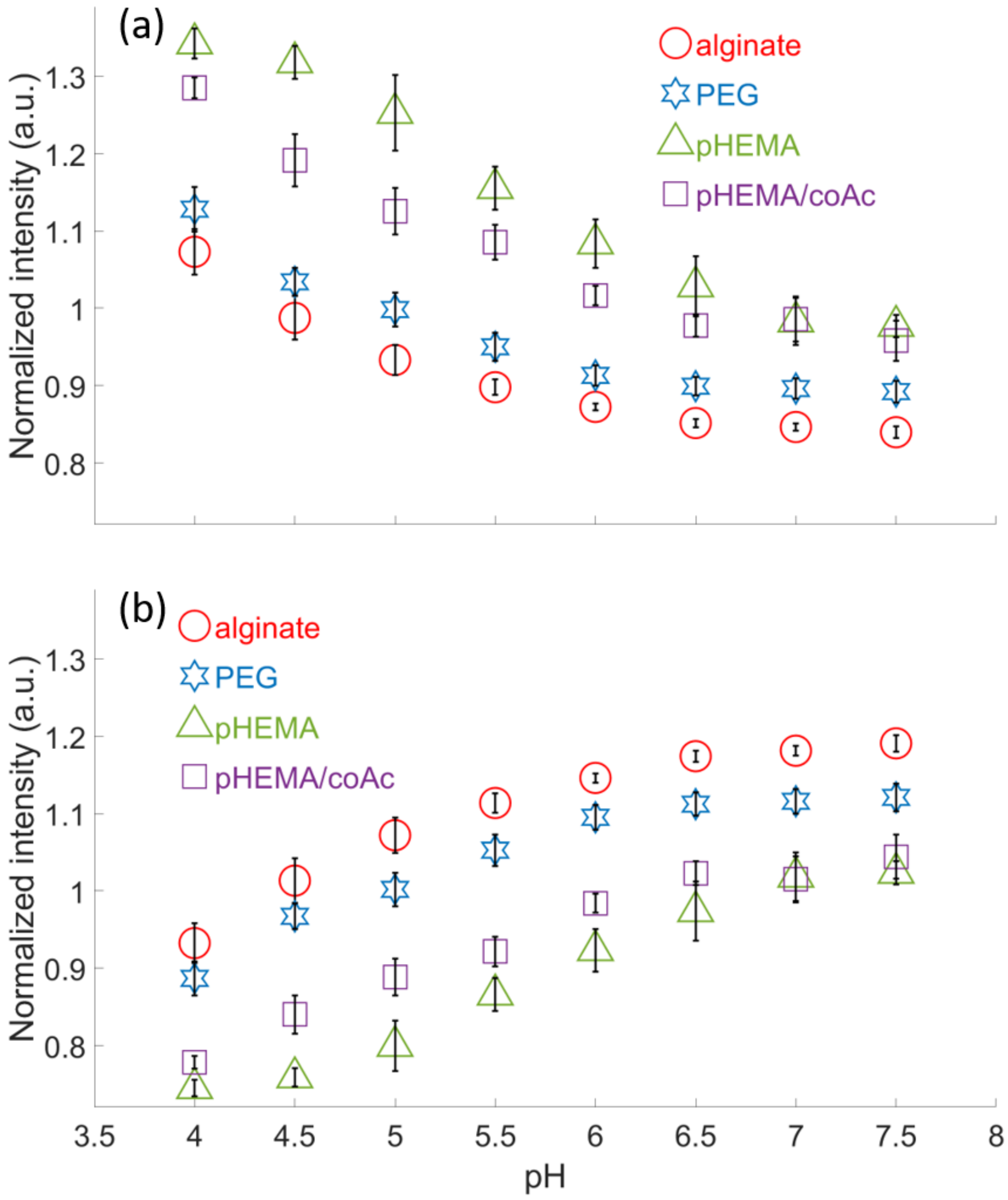


**Fig. S13** Raman reference peak intensity vs static pH level of 5 measurements of 5 punches of the four gel types. (a) 1072 cm$^{-1}$ peak (normalized by 1582 cm$^{-1}$ peak) average with standard deviation error bars. (b) 1582 cm$^{-1}$ peak (normalized by 1072 cm$^{-1}$ peak) average with standard deviation error bars.

**Table S1** 1072 cm$^{-1}$ peak (normalized by 1582 cm$^{-1}$ peak) and 1582 cm$^{-1}$ peak (normalized by 1072 cm$^{-1}$ peak)

| Gel Type | Peak (cm$^{-1}$) | Minimum normalized intensity (a.u.) | Maximum normalized intensity (a.u.) | Range = maximum – minimum (a.u.) | Range/ Minimum (%) |
|---|---|---|---|---|---|

| | | | | | |
|---|---|---|---|---|---|
| Alginate | 1072<br>1582 | 0.840<br>0.933 | 1.073<br>1.191 | 0.233<br>0.258 | 27.800<br>27.713 |
| PEG | 1072<br>1582 | 0.892<br>0.887 | 1.128<br>1.121 | 0.236<br>0.234 | 26.454<br>26.407 |
| pHEMA | 1072<br>1582 | 0.977<br>0.745 | 1.343<br>1.024 | 0.365<br>0.279 | 37.398<br>37.400 |
| pHEMA-coA | 1072<br>1582 | 0.958<br>0.778 | 1.285<br>1.044 | 0.327<br>0.266 | 34.129<br>34.214 |

**3 Dynamic measurements**

The results of the 12 flow cell studies are presented in Fig S14-25. There are three plots for each gel labeled series 1-3. The two pH-sensitive peaks, 1430 $cm^{-1}$ and 1702 $cm^{-1}$ are measured every two minutes and tracked over time as pH in the flow cell is changed nine times. While all the gels show reversibility and repeatability in the 12 figures, some gels are better than others. Since the MBA is more sensitive in an acidic environment, the figures show that the time alotted may be insufficient for the sensors to achieve steady-state at pH 4.0. See, for example, the case of alginate in Fig. S16 where the first pH 4.0 segment (leftmost red segment) does appear to level off for both curves, but that this is achieved to a lesser degree in the subsequent pH4 segments (center and rightmost red segments).

While the alginate response was smooth over time (Fig. S14-S16), noise was present in two out of three of the PEG series (compare Fig. S17 to Figs. S18 and S19).

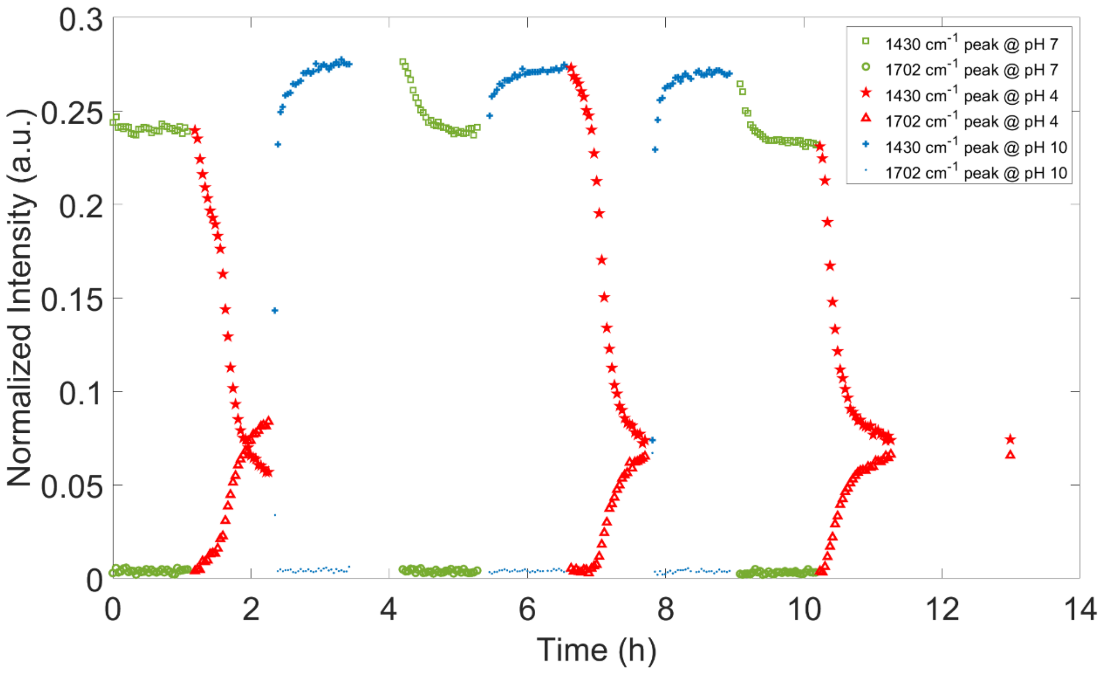


**Fig. S14** Alginate series 1 flow cell study. At pH 7.0, the normalized intensities at 1430 cm$^{-1}$ and 1702 cm$^{-1}$ are shown using the symbol of a square and a circle, respectively. At pH 4.0, the normalized intensities at 1430 cm$^{-1}$ and 1702 cm$^{-1}$ are shown using the symbol of a star and a triangle, respectively. At pH 10.0, the normalized intensities at 1430 cm$^{-1}$ and 1702 cm$^{-1}$, are shown using the symbol of a plus sign and a period, respectively.

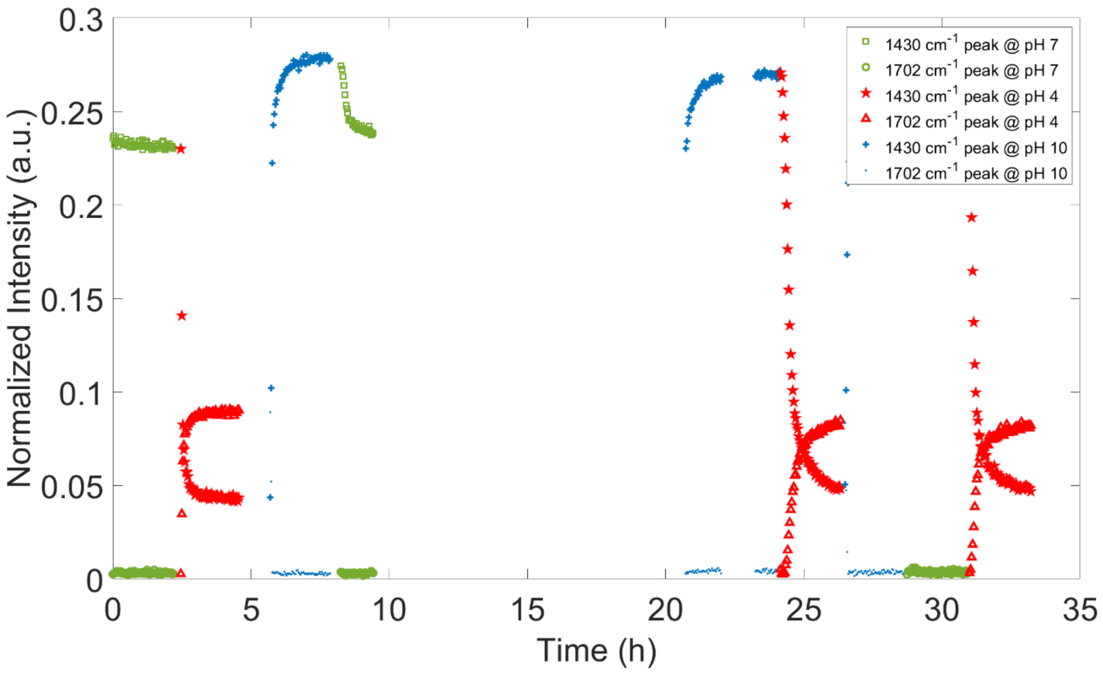

**Fig. S15** Alginate series 2 flow cell study. . At pH 7.0, the normalized intensities at 1430 $cm^{-1}$ and 1702 $cm^{-1}$ are shown using the symbol of a square and a circle, respectively. At pH 4.0, the normalized intensities at 1430 $cm^{-1}$ and 1702 $cm^{-1}$ are shown using the symbol of a star and a triangle, respectively. At pH 10.0, the normalized intensities at 1430 $cm^{-1}$ and 1702 $cm^{-1}$, are shown using the symbol of a plus sign and a period, respectively.

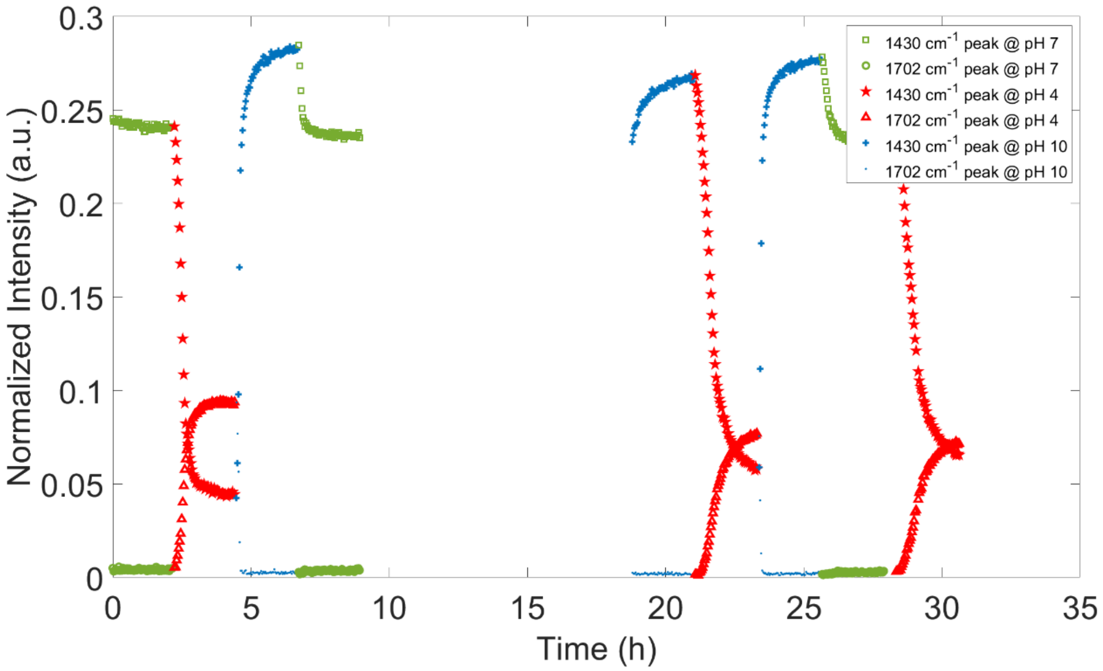


**Fig. S16** Alginate series 3 flow cell study. . At pH 7.0, the normalized intensities at 1430 $cm^{-1}$ and 1702 $cm^{-1}$ are shown using the symbol of a square and a circle, respectively. At pH 4.0, the normalized intensities at 1430 $cm^{-1}$ and 1702 $cm^{-1}$ are shown using the symbol of a star and a triangle, respectively. At pH 10.0, the normalized intensities at 1430 $cm^{-1}$ and 1702 $cm^{-1}$, are shown using the symbol of a plus sign and a period, respectively.

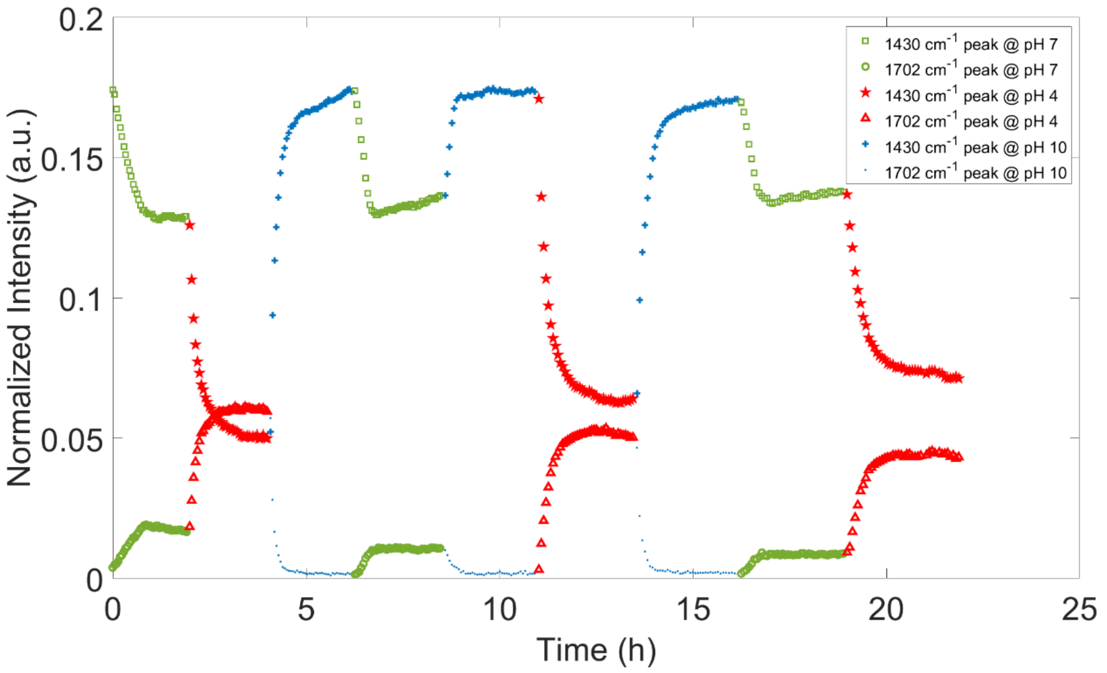


**Fig. S17** PEG series 1 flow cell study. . At pH 7.0, the normalized intensities at 1430 cm$^{-1}$ and 1702 cm$^{-1}$ are shown using the symbol of a square and a circle, respectively. At pH 4.0, the normalized intensities at 1430 cm$^{-1}$ and 1702 cm$^{-1}$ are shown using the symbol of a star and a triangle, respectively. At pH 10.0, the normalized intensities at 1430 cm$^{-1}$ and 1702 cm$^{-1}$, are shown using the symbol of a plus sign and a period, respectively.

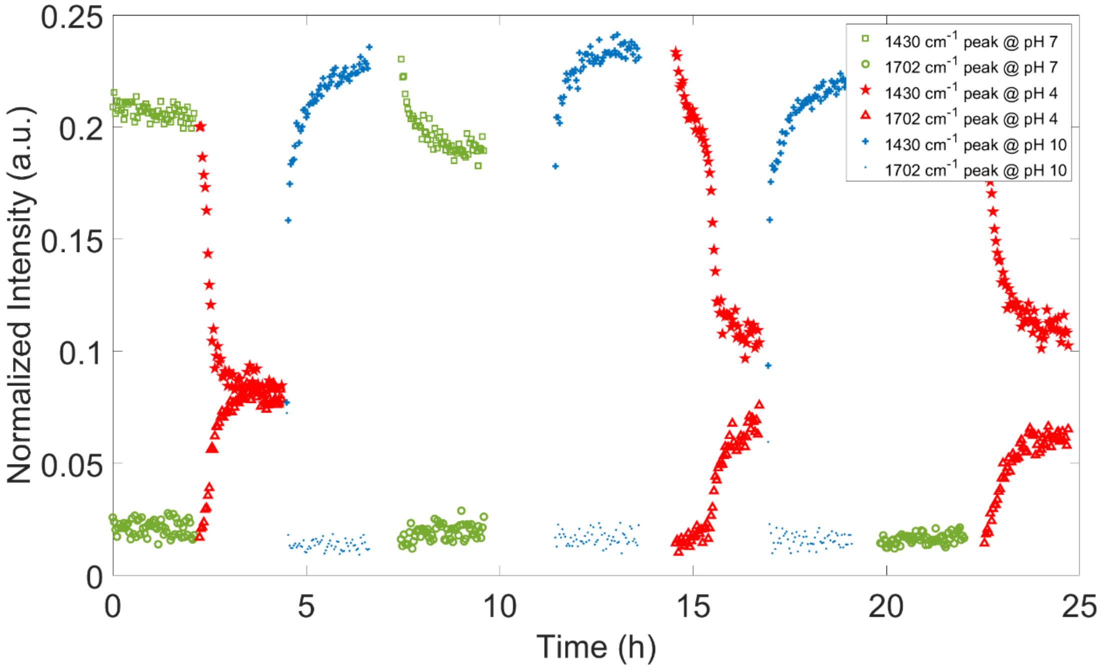


**Fig. S18** PEG series 2 flow cell study. . At pH 7.0, the normalized intensities at 1430 cm$^{-1}$ and 1702 cm$^{-1}$ are shown using the symbol of a square and a circle, respectively. At pH 4.0, the normalized intensities at 1430 cm$^{-1}$ and 1702

cm$^{-1}$ are shown using the symbol of a star and a triangle, respectively. At pH 10.0, the normalized intensities at 1430 cm$^{-1}$ and 1702 cm$^{-1}$, are shown using the symbol of a plus sign and a period, respectively.

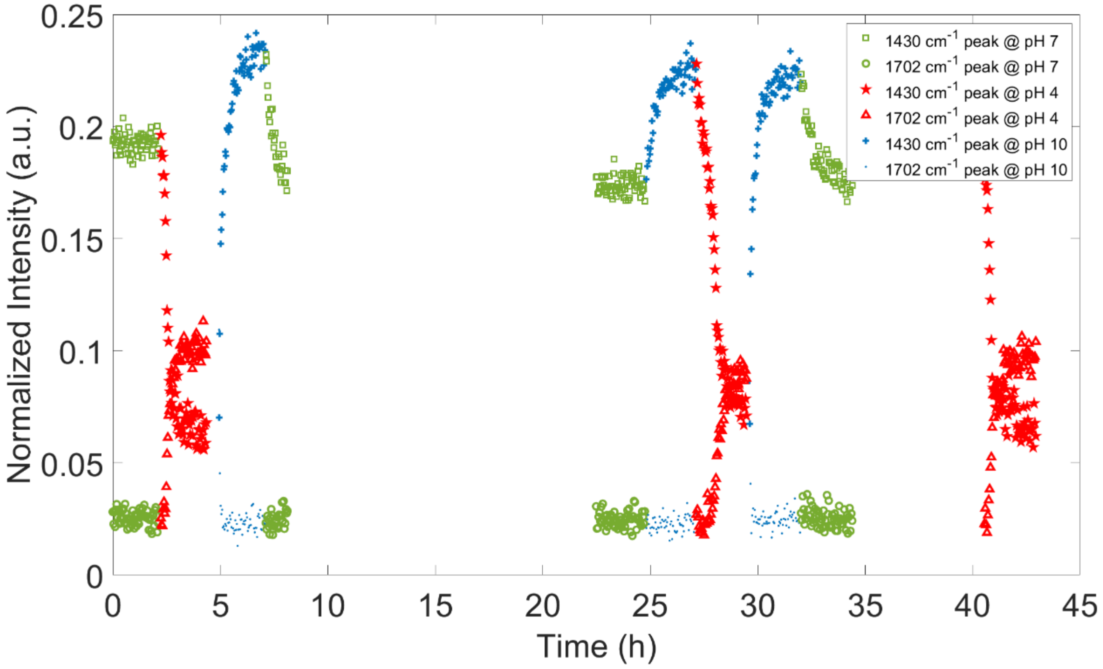


**Fig. S19** PEG series 3 flow cell study. . At pH 7.0, the normalized intensities at 1430 cm$^{-1}$ and 1702 cm$^{-1}$ are shown using the symbol of a square and a circle, respectively. At pH 4.0, the normalized intensities at 1430 cm$^{-1}$ and 1702 cm$^{-1}$ are shown using the symbol of a star and a triangle, respectively. At pH 10.0, the normalized intensities at 1430 cm$^{-1}$ and 1702 cm$^{-1}$, are shown using the symbol of a plus sign and a period, respectively.

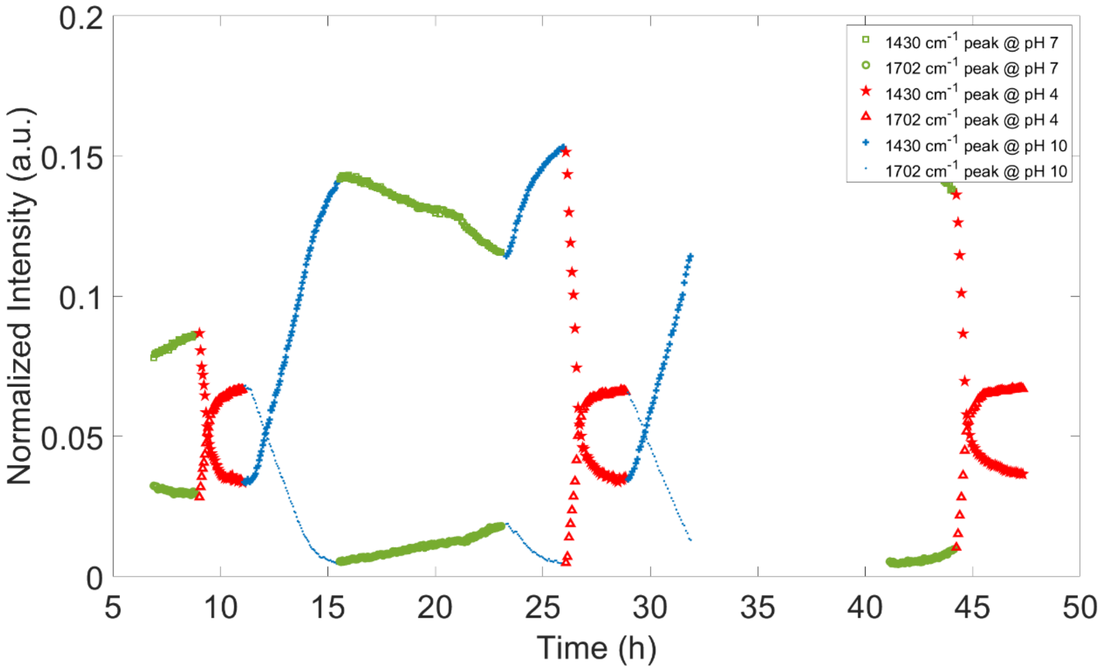

**Fig. S20** pHEMA series 1 flow cell study. . At pH 7.0, the normalized intensities at 1430 $cm^{-1}$ and 1702 $cm^{-1}$ are shown using the symbol of a square and a circle, respectively. At pH 4.0, the normalized intensities at 1430 $cm^{-1}$ and 1702 $cm^{-1}$ are shown using the symbol of a star and a triangle, respectively. At pH 10.0, the normalized intensities at 1430 $cm^{-1}$ and 1702 $cm^{-1}$, are shown using the symbol of a plus sign and a period, respectively.

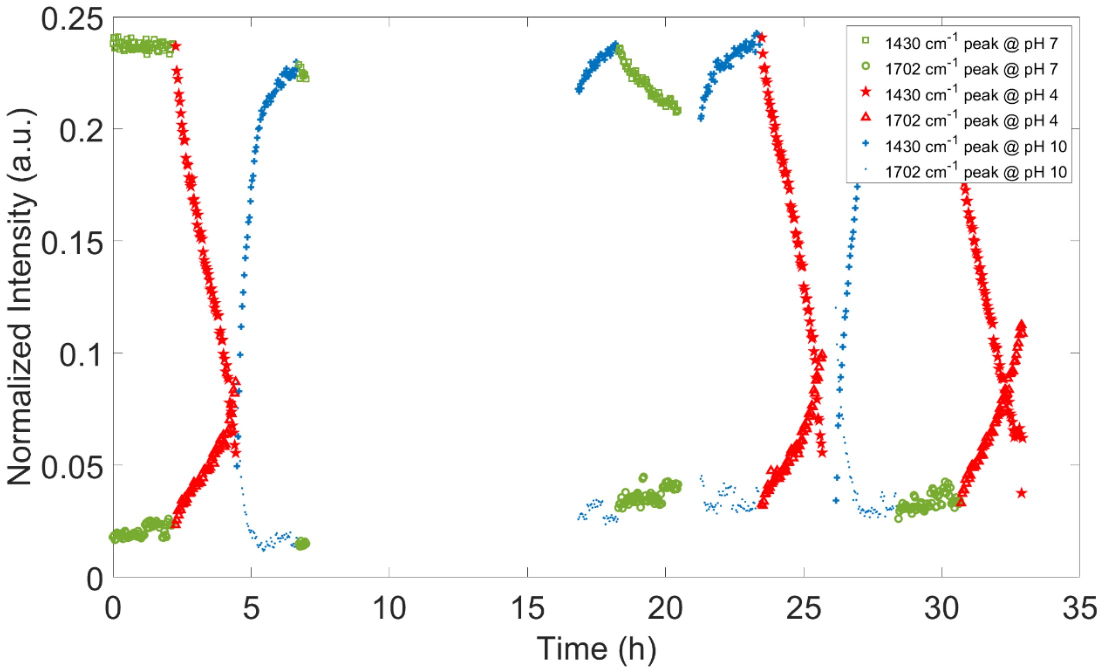


**Fig. S21** pHEMA series 2 flow cell study. . At pH 7.0, the normalized intensities at 1430 $cm^{-1}$ and 1702 $cm^{-1}$ are shown using the symbol of a square and a circle, respectively. At pH 4.0, the normalized intensities at 1430 $cm^{-1}$ and 1702 $cm^{-1}$ are shown using the symbol of a star and a triangle, respectively. At pH 10.0, the normalized intensities at 1430 $cm^{-1}$ and 1702 $cm^{-1}$, are shown using the symbol of a plus sign and a period, respectively.

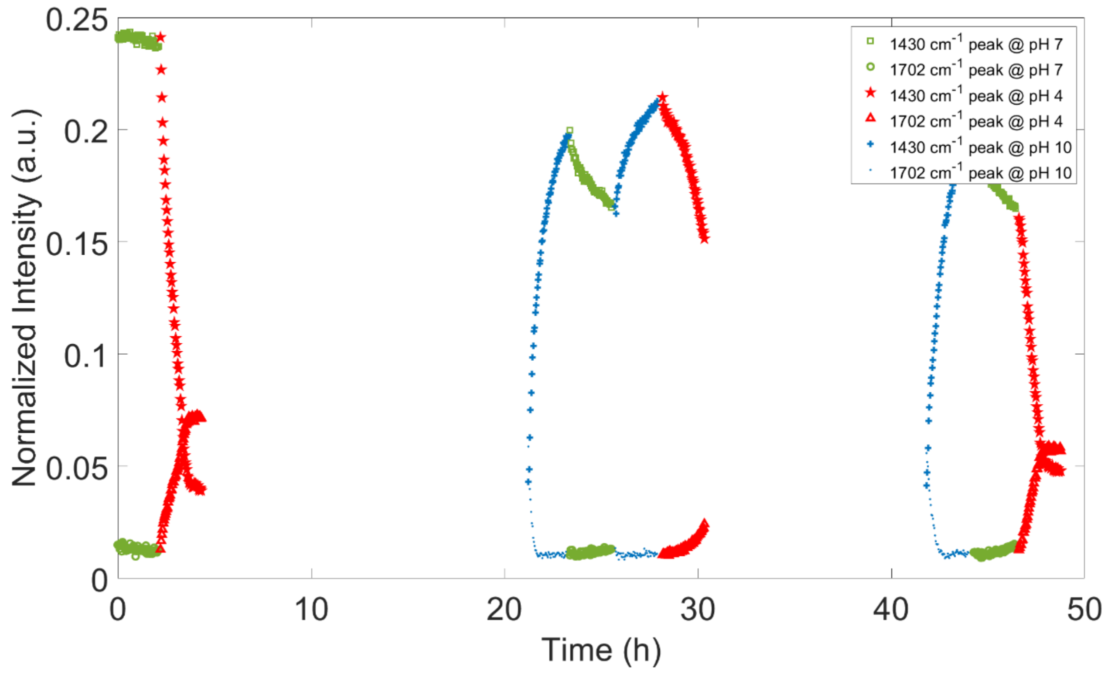


**Fig. S22** pHEMA series 3 flow cell study. . At pH 7.0, the normalized intensities at 1430 $cm^{-1}$ and 1702 $cm^{-1}$ are shown using the symbol of a square and a circle, respectively. At pH 4.0, the normalized intensities at 1430 $cm^{-1}$ and 1702 $cm^{-1}$ are shown using the symbol of a star and a triangle, respectively. At pH 10.0, the normalized intensities at 1430 $cm^{-1}$ and 1702 $cm^{-1}$, are shown using the symbol of a plus sign and a period, respectively.

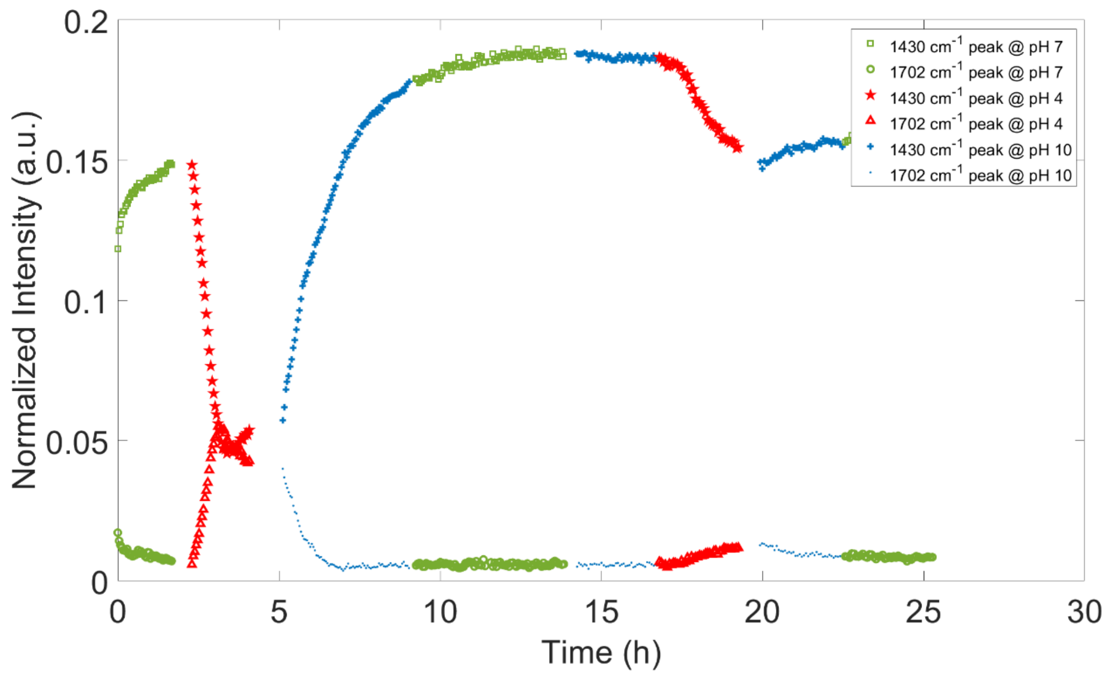


**Fig. S23** pHEMA-coA series 1 flow cell study. . At pH 7.0, the normalized intensities at 1430 $cm^{-1}$ and 1702 $cm^{-1}$ are shown using the symbol of a square and a circle, respectively. At pH 4.0, the normalized intensities at 1430 $cm^{-1}$

and 1702 cm$^{-1}$ are shown using the symbol of a star and a triangle, respectively. At pH 10.0, the normalized intensities at 1430 cm$^{-1}$ and 1702 cm$^{-1}$, are shown using the symbol of a plus sign and a period, respectively.

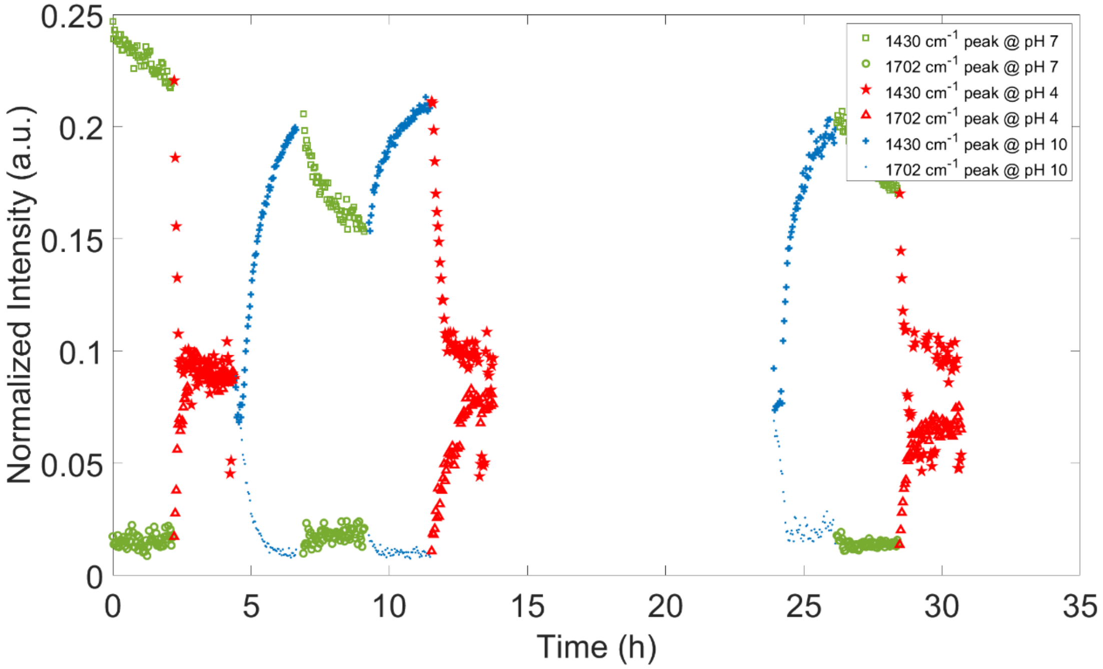


**Fig. S24** pHEMA-coA series 2 flow cell study. . At pH 7.0, the normalized intensities at 1430 cm$^{-1}$ and 1702 cm$^{-1}$ are shown using the symbol of a square and a circle, respectively. At pH 4.0, the normalized intensities at 1430 cm$^{-1}$ and 1702 cm$^{-1}$ are shown using the symbol of a star and a triangle, respectively. At pH 10.0, the normalized intensities at 1430 cm$^{-1}$ and 1702 cm$^{-1}$, are shown using the symbol of a plus sign and a period, respectively.

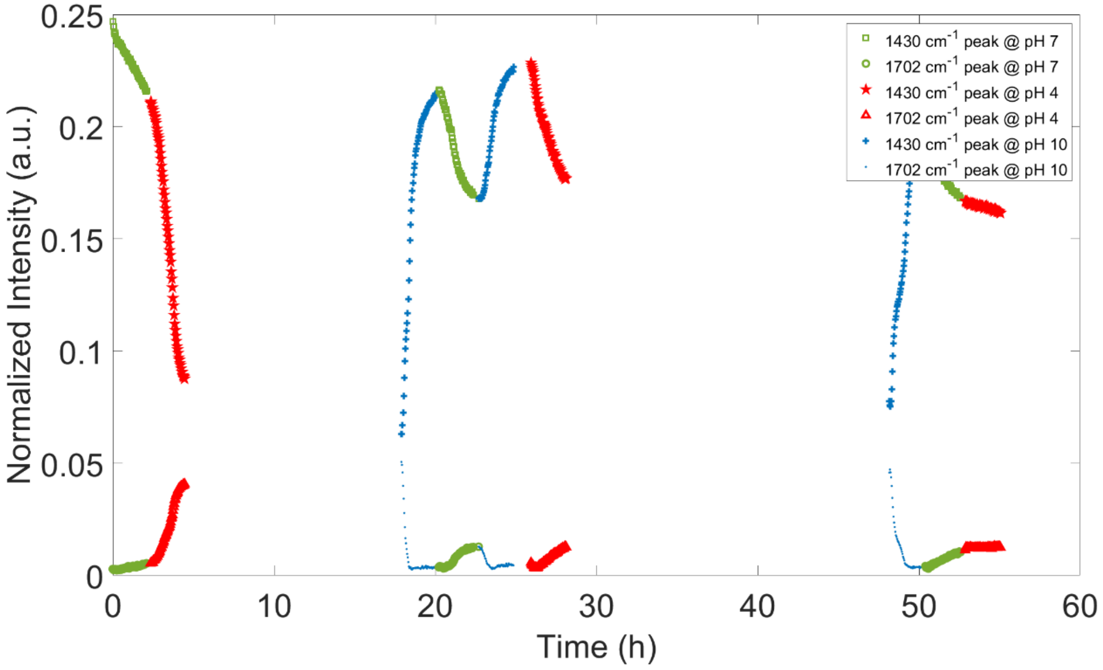


**Fig. S25** pHEMA-coA series 3 flow cell study. . At pH 7.0, the normalized intensities at 1430 $cm^{-1}$ and 1702 $cm^{-1}$ are shown using the symbol of a square and a circle, respectively. At pH 4.0, the normalized intensities at 1430 $cm^{-1}$ and 1702 $cm^{-1}$ are shown using the symbol of a star and a triangle, respectively. At pH 10.0, the normalized intensities at 1430 $cm^{-1}$ and 1702 $cm^{-1}$, are shown using the symbol of a plus sign and a period, respectively.

### 3.1 Curve-fitting

Since the hydrogels were slow to attain the steady-state intensity value each time the pH level was changed, time constants were determined by fitting exponential curves to the latter portion of each segment in Fig. S14-S25. The results of the curve fitting for twelve flow cell studies are presented in Fig. S26-S38, including two figures (Fig. S26 and S27) for alginate series 1 to show the difference between using the last 29 points (Fig. S26) and the last 20 points of each segment (Fig. S27). The fitted curves are overlaid as solid lines on the marker symbols indicating the measured values. Note how the inflection points in the curves of the first pH 4.0 segments (leftmost red segment) are actually better fit by the lower number of points. Also note how the second pH 4.0 segment (center red segment) would be better fit with even fewer than 20 points because of its inflection points. The remainder of the plots used the last 29 points of each segment to fit the curves.

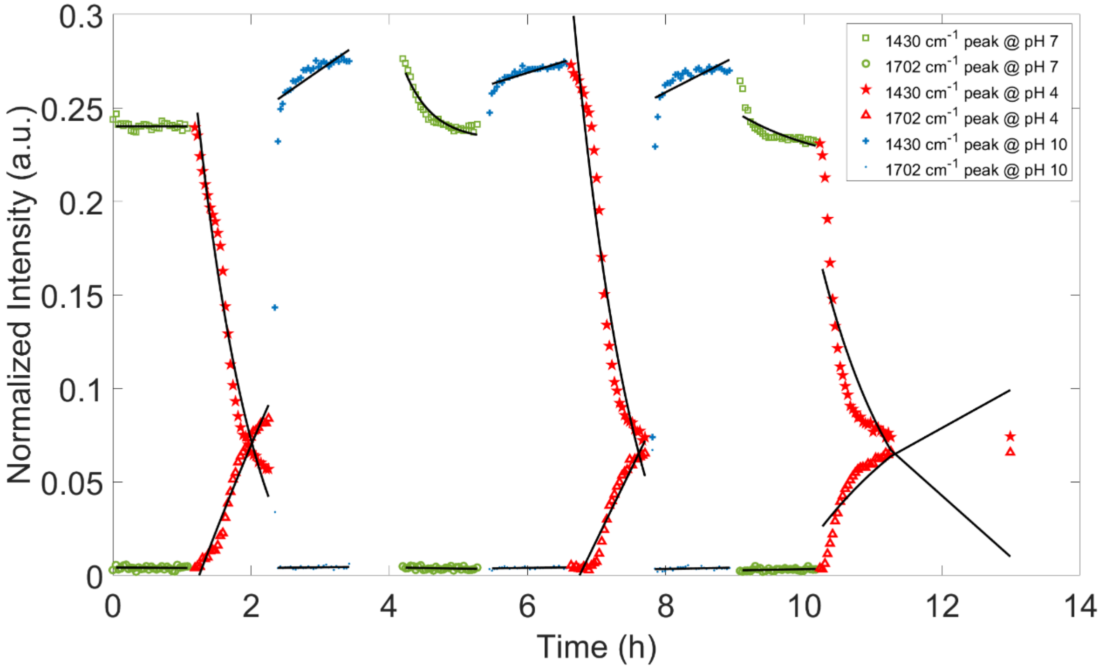


**Fig. S26** Alginate series 1 flow cell study using last 29 points per pH segment. The fitted curves are overlaid as solid lines on the measured values drawn with marker symbols defined in the legend.

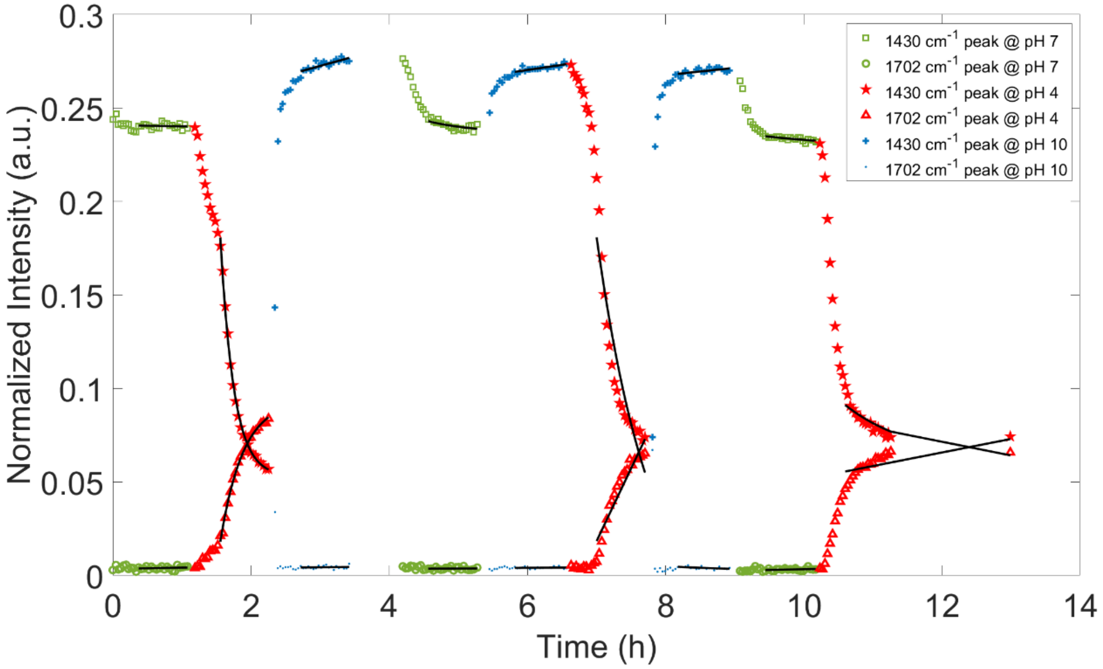


**Fig. S27** Alginate series 1 flow cell study using last 20 points per pH segment. The fitted curves are overlaid as solid lines on the measured values drawn with marker symbols defined in the legend.

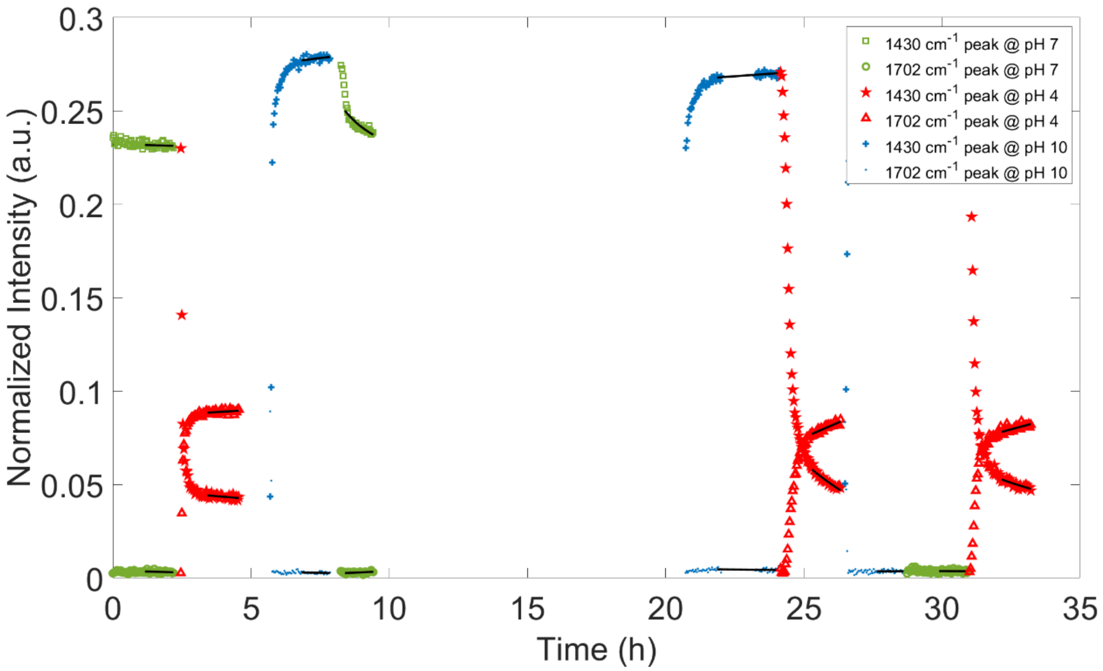


**Fig. S28** Alginate series 2 flow cell study using last 29 points per pH segment. The fitted curves are overlaid as solid lines on the measured values drawn with marker symbols defined in the legend.

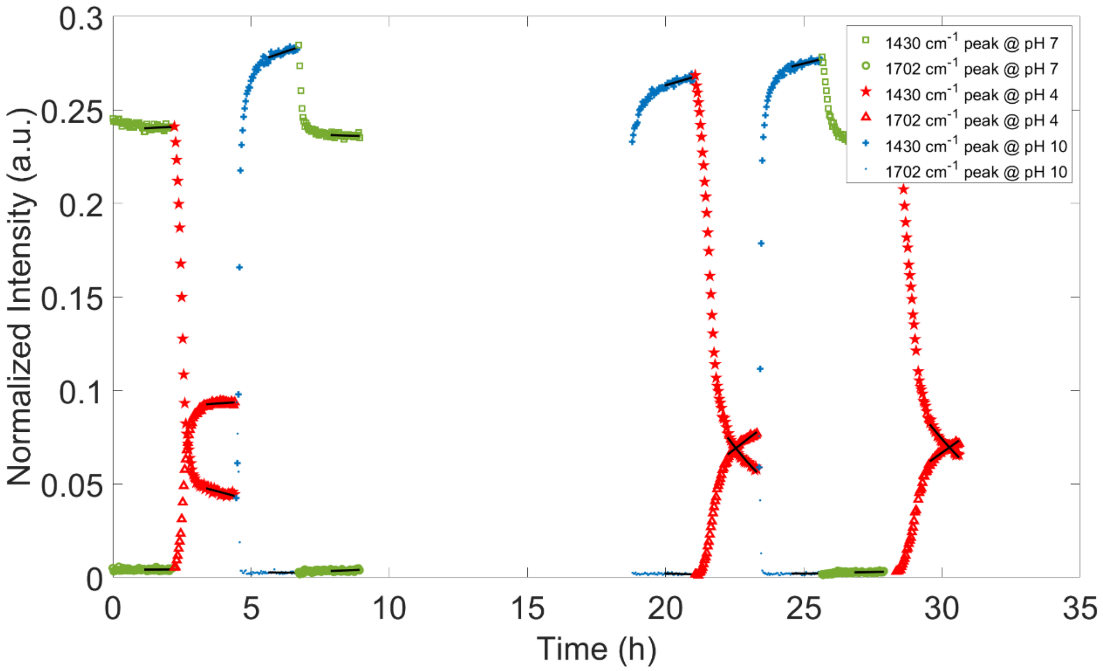


**Fig. S29** Alginate series 3 flow cell study using last 29 points per pH segment. The fitted curves are overlaid as solid lines on the measured values drawn with marker symbols defined in the legend.

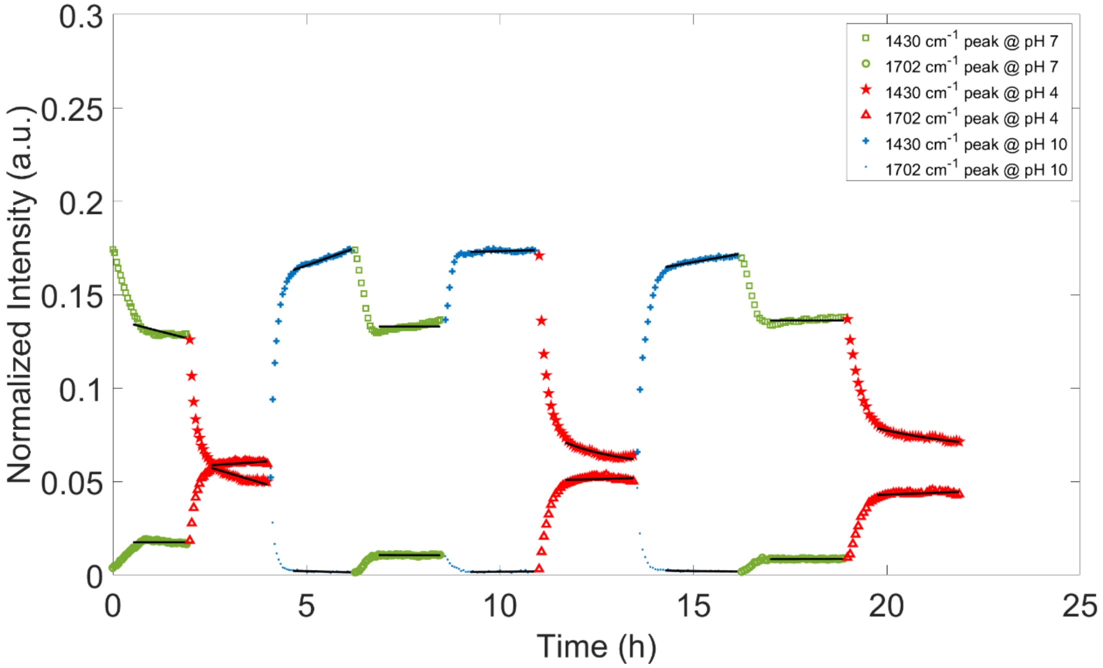


**Fig. S30** PEG series 1 flow cell study using last 29 points per pH segment. The fitted curves are overlaid as solid lines on the measured values drawn with marker symbols defined in the legend.

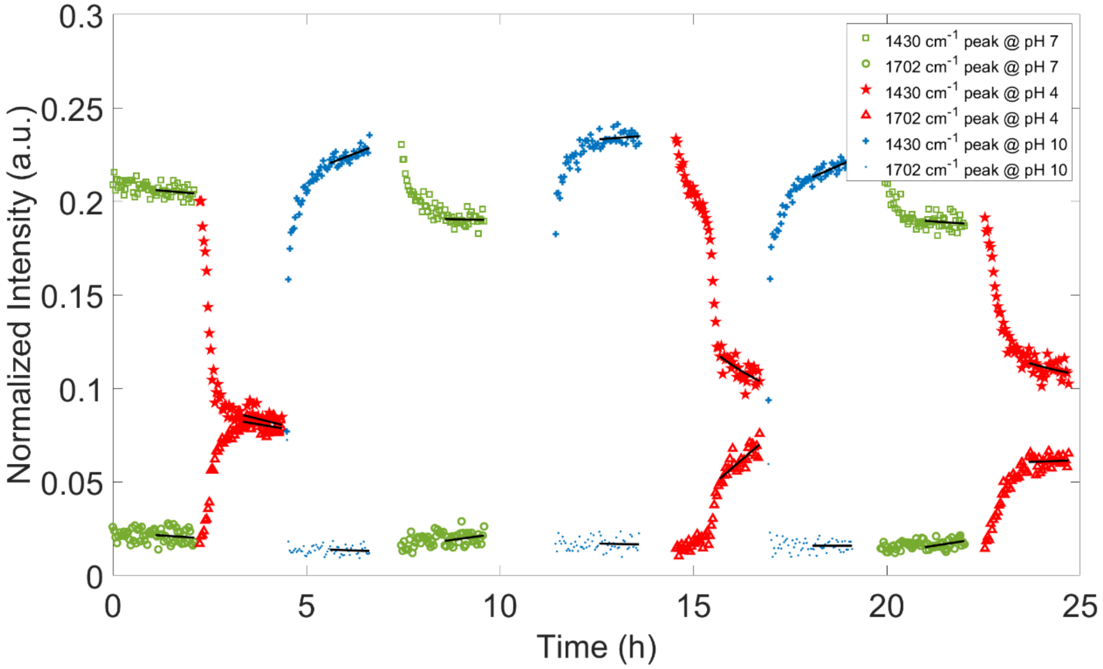


**Fig. S31** PEG series 2 flow cell study using last 29 points per pH segment. The fitted curves are overlaid as solid lines on the measured values drawn with marker symbols defined in the legend.

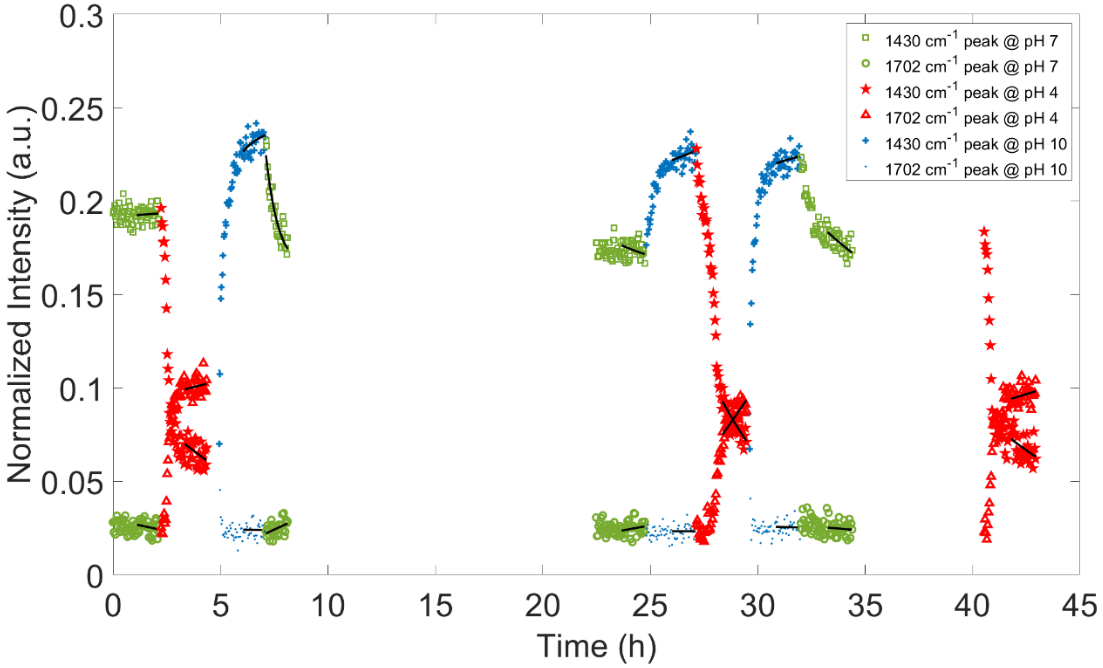


**Fig. S32** PEG series 3 flow cell study using last 29 points per pH segment. The fitted curves are overlaid as solid lines on the measured values drawn with marker symbols defined in the legend.

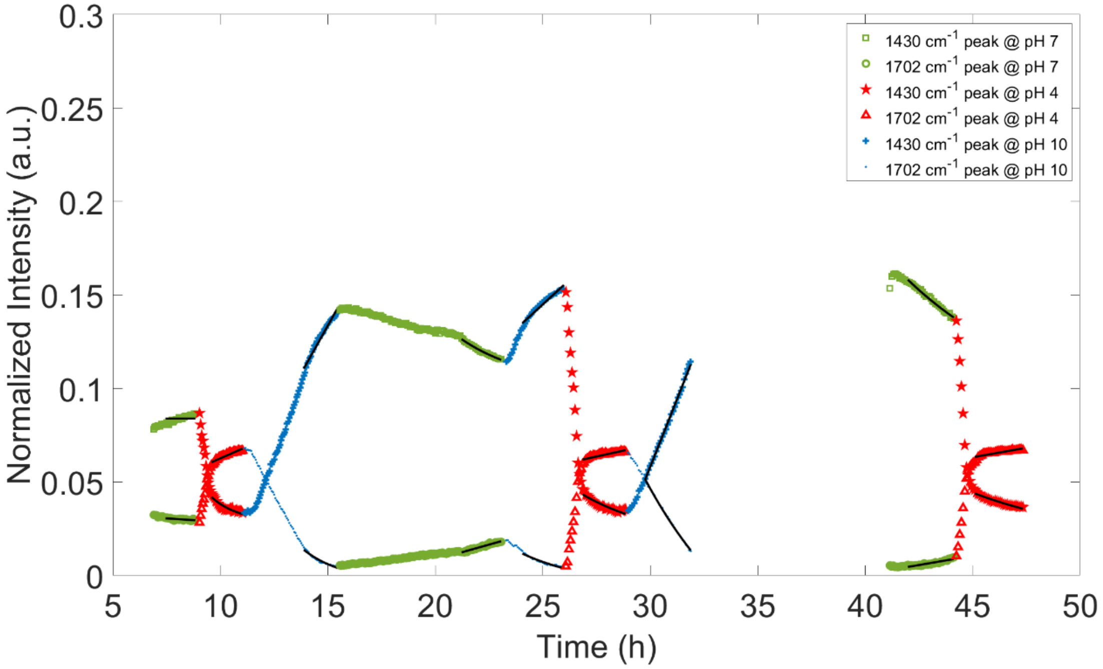


**Fig. S33** pHEMA series 1 flow cell study using last 29 points per pH segment. The fitted curves are overlaid as solid lines on the measured values drawn with marker symbols defined in the legend.

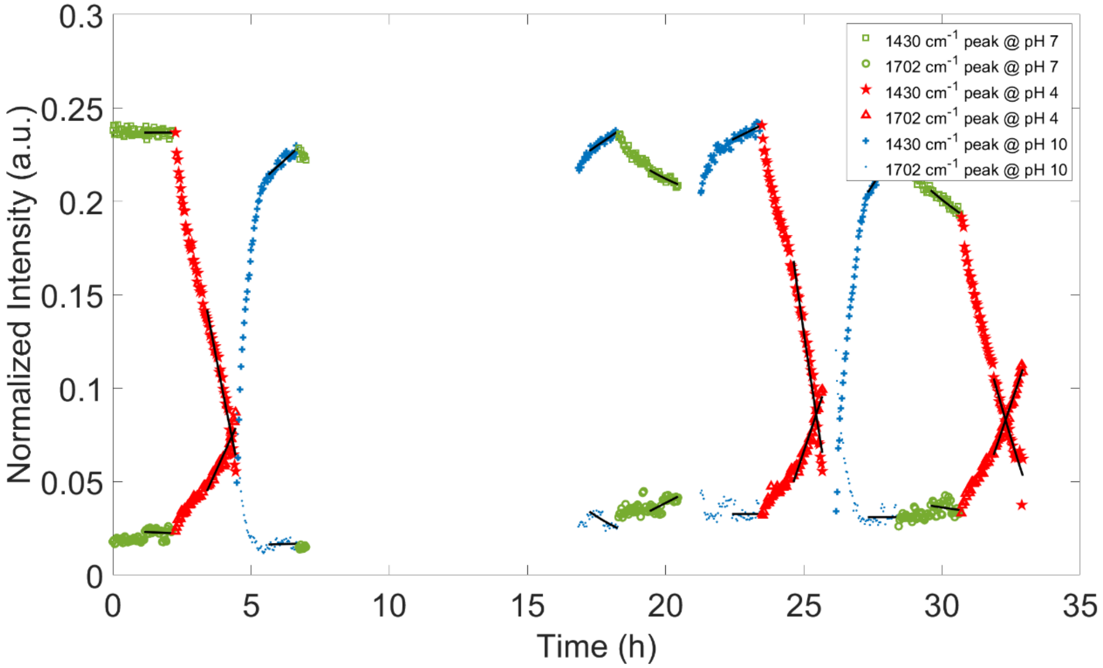


**Fig. S34** pHEMA series 2 flow cell study using last 29 points per pH segment. The fitted curves are overlaid as solid lines on the measured values drawn with marker symbols defined in the legend.

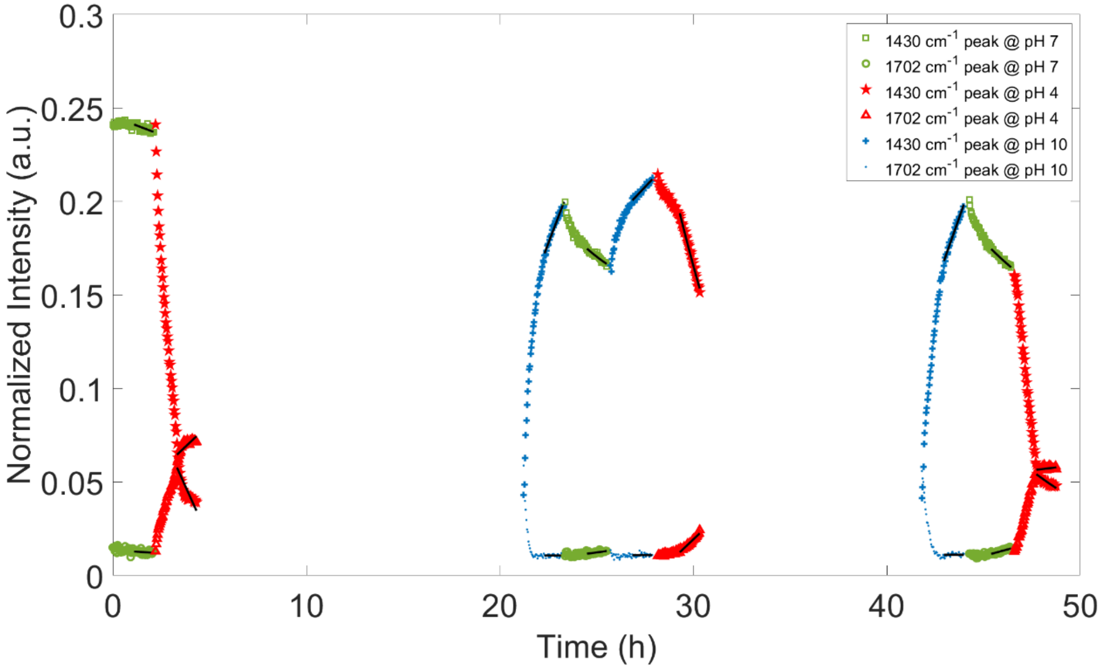


**Fig. S35** pHEMA series 3 flow cell study using last 29 points per pH segment. The fitted curves are overlaid as solid lines on the measured values drawn with marker symbols defined in the legend.

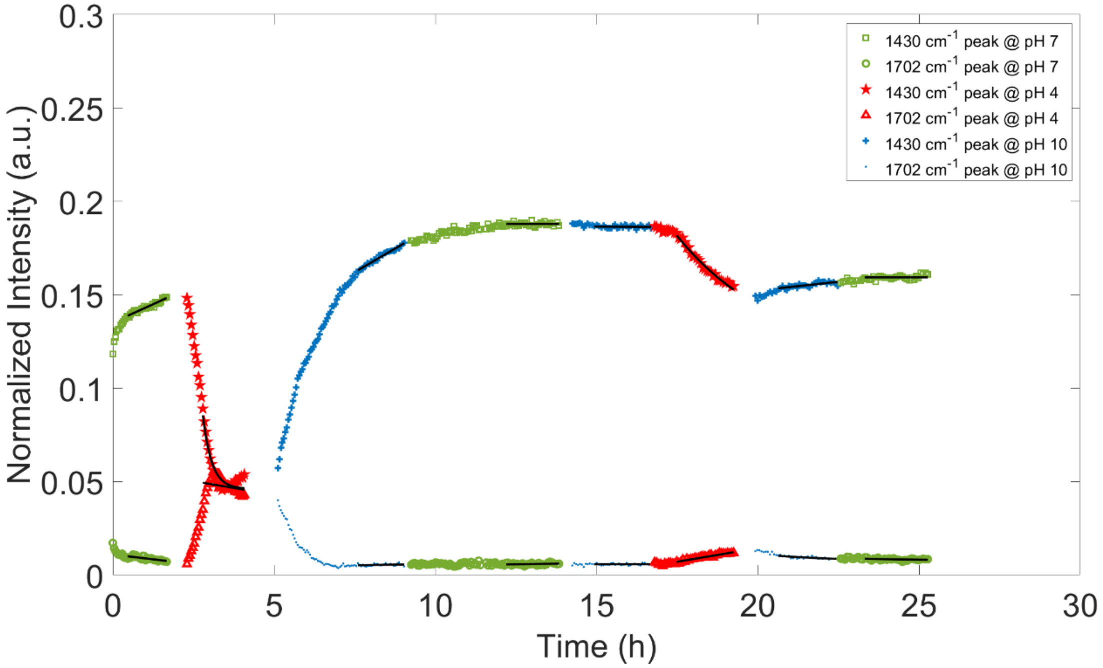


**Fig. S36** pHEMA-coA series 1 flow cell study using last 29 points per pH segment. The fitted curves are overlaid as solid lines on the measured values drawn with marker symbols defined in the legend.

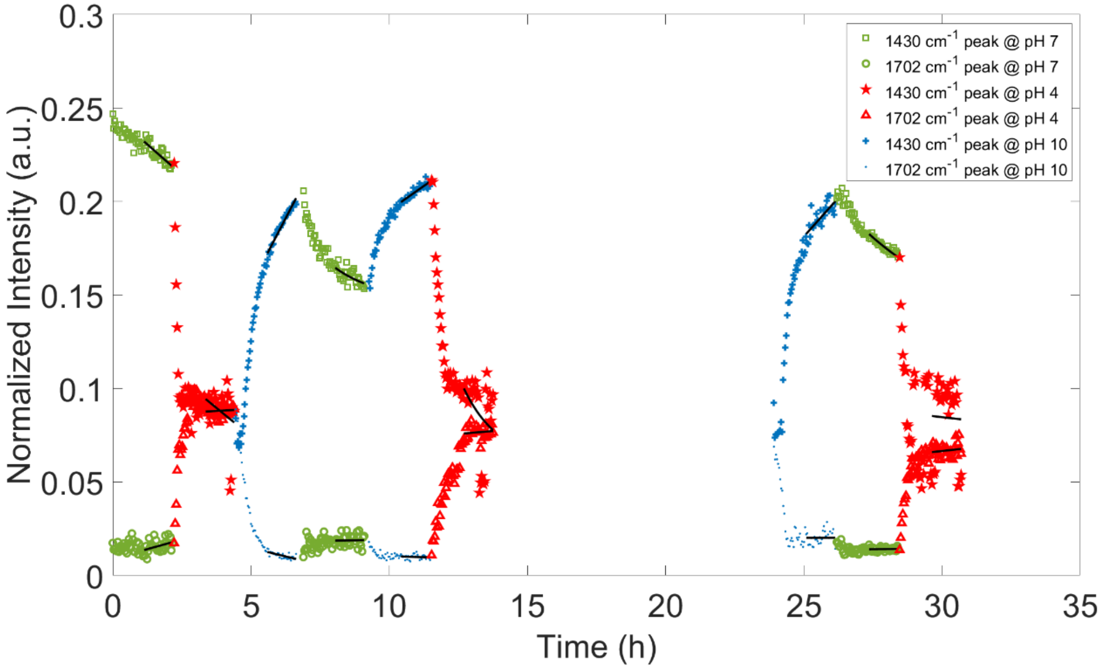


**Fig. S37** pHEMA-coA series 2 flow cell study using last 29 points per pH segment. The fitted curves are overlaid as solid lines on the measured values drawn with marker symbols defined in the legend.

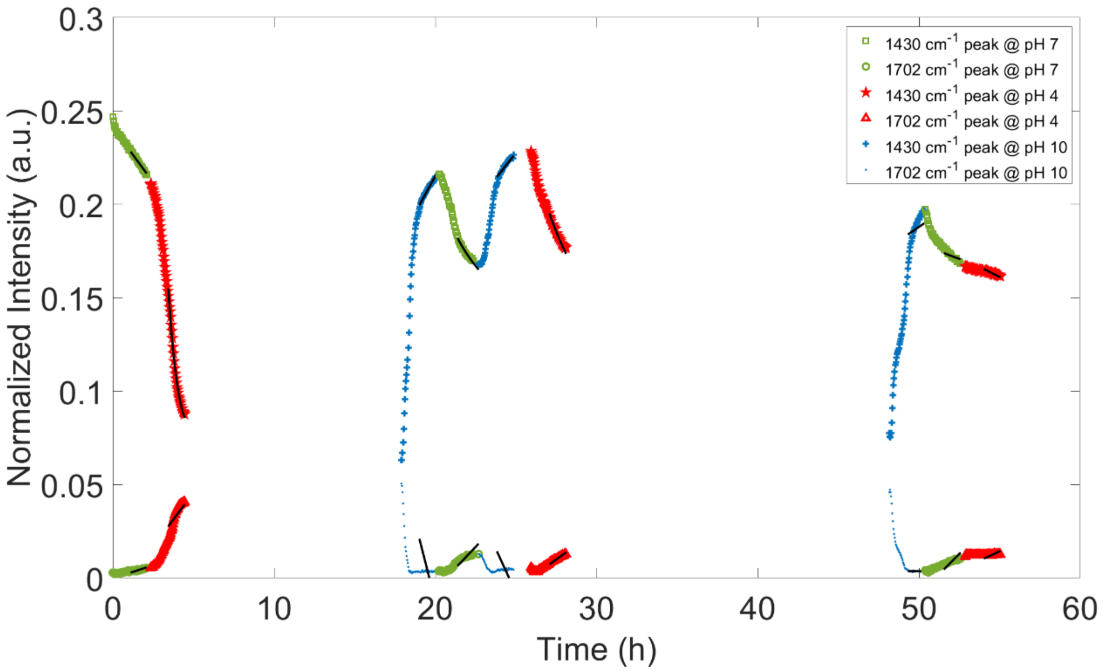


**Fig. S38** pHEMA-coA series 3 flow cell study using last 29 points per pH segment. The fitted curves are overlaid as solid lines on the measured values drawn with marker symbols defined in the legend.

### *3.2 Time constants*

The results of the curve-fitting are a set of time constants at three different pH levels are shown in Fig. S39-S41. These plots expand the resolution Fig. 7 (of the main manuscript) which shows the time constants for all pH levels on a single plot. Examining these three plots, it can be seen that PEG is the only gel that appears in the "< 10 min" bin at all three pH levels, while pHEMA appears in this bin at pH 7.0 and pH 10.0, alginate appears in this bin at pH 7.0 and pHEMA-coA does not appear in this bin at any pH level.

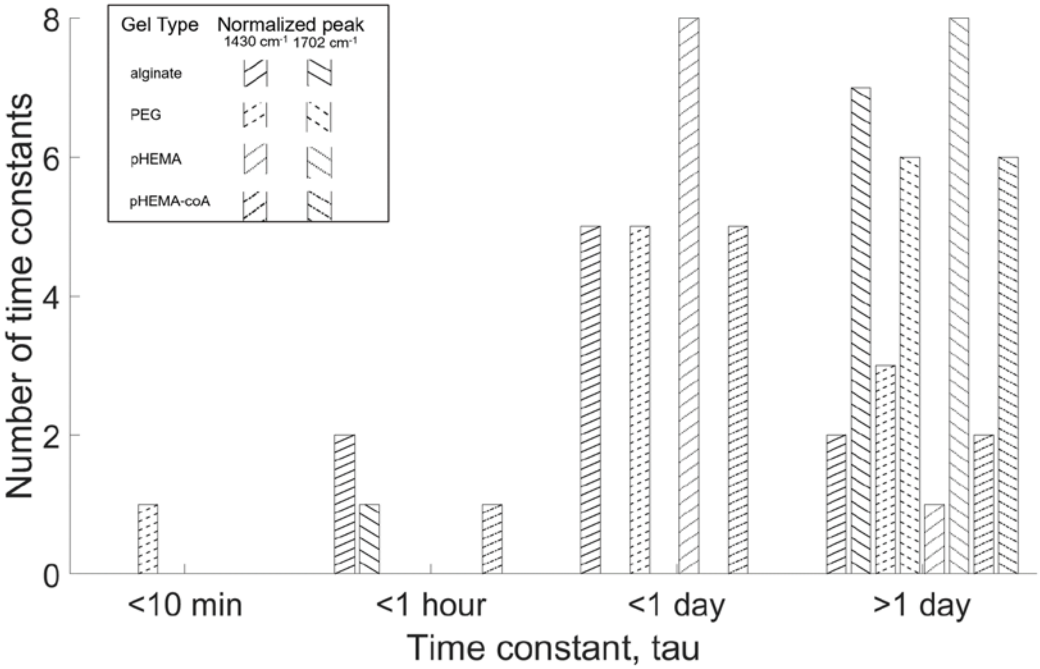


**Fig. S39** Distribution of time constants for the normalized intensities of two pH-sensitive peaks, at 1430 $cm^{-1}$ and 1702 $cm^{-1}$, for all pH 4.0 segments, all series and all gel types (alginate, PEG, pHEMA and pHEMA-coA). 8 different fill patterns are defined in the legend to represent the 8 cases.

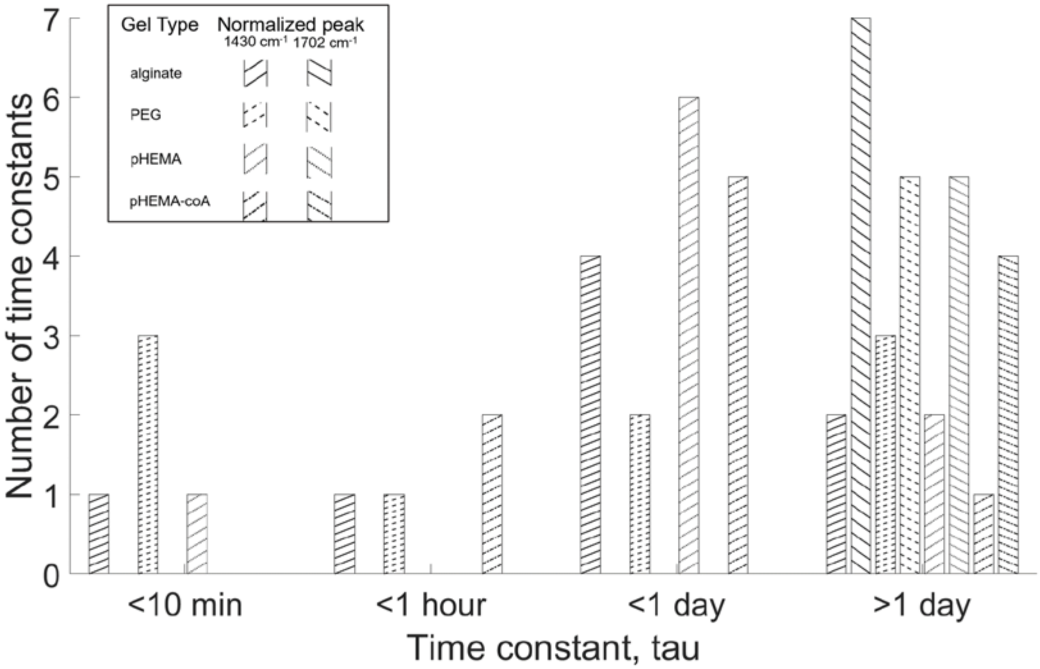


**Fig. S40** Distribution of time constants for the normalized intensities of two pH-sensitive peaks, at 1430 $cm^{-1}$ and 1702 $cm^{-1}$, for all pH 7.0 segments, all series and all gel types (alginate, PEG, pHEMA and pHEMA-coA). 8 different fill patterns are defined in the legend to represent the 8 cases.

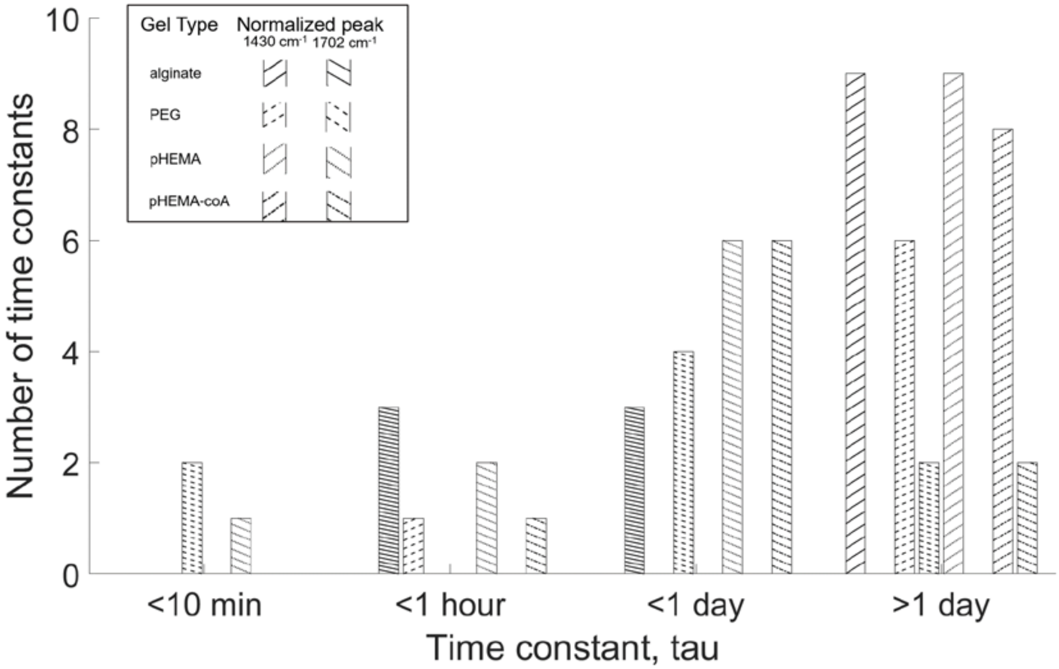

**Fig. S41** Distribution of time constants for the normalized intensities of two pH-sensitive peaks, at 1430 $cm^{-1}$ and 1702 $cm^{-1}$, for all pH 10.0 segments, all series and all gel types (alginate, PEG, pHEMA and pHEMA-coA). 8 different fill patterns are defined in the legend to represent the 8 cases.

## 4 Consistency of static and dynamic measurements

In Fig. 8 and 9 (of the main manuscript), the standard deviation of the measurements was used for the error bars on the bar graphs. The sample size was 5 for the static measurements, since only the final average of each gel punch was used, and 9 for the dynamic measurements, since only the average of the three matching pH levels over the three series was used. By returning to the full set of raw spectra that were collected, it was possible to calculate the standard deviation with larger sample sizes. Since five raw spectra were collected for each of the five averaged spectra per punch, the new sample size for the static measurements increased to 125 (from 5). Similarly, since five raw spectra were collected for each of the average spectra in the dynamic measurements, the new sample size for the dynamic measurements increased to 45 (from 9).

The magnitude of the standard deviation (shown as the error bars in Fig. 8 and 9) as a function of sample size for both the static and dynamic datasets is shown in Fig. S42. The numbered cases on the horizontal axis are defined in Table S2. Fig. S42 shows that while increasing the sample size for the static measurements (from blue plus sign to red circle) does reduce the variance significantly (case 7 shows a reduction of more than 7x), it does not help to reduce the variance of the dynamic measurements (from black square to green asterisk).

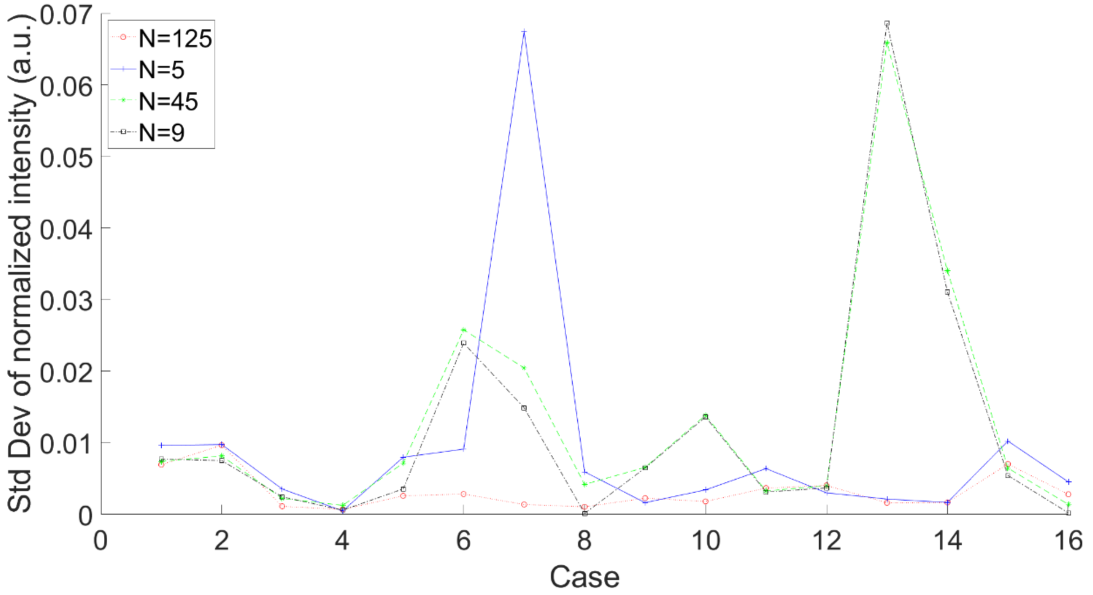


**Fig. S42** Effect of increasing sample size on magnitude of standard deviation. Blue and red represent the static measurement case; black and green represent the dynamic measurement case.

**Table S2** Case description for Fig. S42

| Case | Gel | pH | Peak | *STATIC* | | | | | | *DYNAMIC* | | | | | |
|---|---|---|---|---|---|---|---|---|---|---|---|---|---|---|---|
| | | | | NEW | | | OLD | | | NEW | | | OLD | | |
| | | | | N | AVG | STD DEV | N | AVG | STD DEV | N | AVG | STD DEV | N | AVG | STD DEV |
| 1 | alg | 4 | 1430 | 125 | 0.050614 | 0.006947 | 5 | 0.05739 | 0.009642 | 45 | 0.043507 | 0.007413 | 9 | 0.043321 | 0.007735 |
| 2 | alg | 4 | 1702 | 125 | 0.077846 | 0.009672 | 5 | 0.06724 | 0.009762 | 45 | 0.068571 | 0.008159 | 9 | 0.069256 | 0.007517 |
| 3 | alg | 7 | 1430 | 125 | 0.207319 | 0.001134 | 5 | 0.20616 | 0.003545 | 45 | 0.219924 | 0.002228 | 9 | 0.219979 | 0.002454 |
| 4 | alg | 7 | 1702 | 125 | 0.003363 | 0.000689 | 5 | 0.00379 | 0.000482 | 45 | 0.001915 | 0.001259 | 9 | 0.001152 | 0.000592 |
| 5 | peg | 4 | 1430 | 125 | 0.065292 | 0.002591 | 5 | 0.0612 | 0.007982 | 45 | 0.066678 | 0.007152 | 9 | 0.045803 | 0.003556 |
| 6 | peg | 4 | 1702 | 125 | 0.06679 | 0.002842 | 5 | 0.06608 | 0.009121 | 45 | 0.094326 | 0.025783 | 9 | 0.09034 | 0.023921 |
| 7 | peg | 7 | 1430 | 125 | 0.139926 | 0.001381 | 5 | 0.08707 | 0.067462 | 45 | 0.163951 | 0.020467 | 9 | 0.1604 | 0.014895 |
| 8 | peg | 7 | 1702 | 125 | 0.014042 | 0.001062 | 5 | 0.00757 | 0.005948 | 45 | 0.017694 | 0.004187 | 9 | 0.012788 | 0.000137 |
| 9 | pHe | 4 | 1430 | 125 | 0.031638 | 0.002262 | 5 | 0.02966 | 0.00164 | 45 | 0.046865 | 0.006567 | 9 | 0.045419 | 0.006474 |
| 10 | pHe | 4 | 1702 | 125 | 0.090542 | 0.001811 | 5 | 0.09058 | 0.003423 | 45 | 0.056905 | 0.013851 | 9 | 0.055791 | 0.013617 |

| | | | | | | | | | | | | | | |
|---|---|---|---|---|---|---|---|---|---|---|---|---|---|---|
| 11 | pHe | 7 | 1430 | 125 | 0.173391 | 0.003672 | 5 | 0.16937 | 0.006402 | 45 | 0.148147 | 0.00328 | 9 | 0.147845 | 0.003123 |
| 12 | pHe | 7 | 1702 | 125 | 0.012681 | 0.004042 | 5 | 0.01564 | 0.003012 | 45 | 0.013596 | 0.003989 | 9 | 0.013051 | 0.003688 |
| 13 | pHC | 4 | 1430 | 125 | 0.045174 | 0.001621 | 5 | 0.04387 | 0.002142 | 45 | 0.14954 | 0.065921 | 8 | 0.149728 | 0.068656 |
| 14 | pHC | 4 | 1702 | 125 | 0.080342 | 0.001659 | 5 | 0.082 | 0.00167 | 45 | 0.011915 | 0.034032 | 9 | 0.01183 | 0.031081 |
| 15 | pHC | 7 | 1430 | 125 | 0.133118 | 0.00704 | 5 | 0.13798 | 0.010244 | 45 | 0.156396 | 0.006468 | 9 | 0.156327 | 0.00544 |
| 16 | pHC | 7 | 1702 | 125 | 0.026508 | 0.002812 | 5 | 0.02236 | 0.004534 | 45 | 0.009377 | 0.001392 | 9 | 0.009675 | 0.00021 |
